# The codon sequences predict protein lifetimes and other parameters of the protein life cycle in the mouse brain

**DOI:** 10.1038/s41598-018-35277-8

**Published:** 2018-11-15

**Authors:** Sunit Mandad, Raza-Ur Rahman, Tonatiuh Pena Centeno, Ramon O. Vidal, Hanna Wildhagen, Burkhard Rammner, Sarva Keihani, Felipe Opazo, Inga Urban, Till Ischebeck, Koray Kirli, Eva Benito, André Fischer, Roya Y. Yousefi, Sven Dennerlein, Peter Rehling, Ivo Feussner, Henning Urlaub, Stefan Bonn, Silvio O. Rizzoli, Eugenio F. Fornasiero

**Affiliations:** 1 Department of Neuro- and Sensory Physiology, University Medical Center Göttingen, Cluster of Excellence Nanoscale Microscopy and Molecular Physiology of the Brain, 37073 Göttingen, Germany; 2 Department of Clinical Chemistry, University Medical Center Göttingen, 37077 Göttingen, Germany; 3 Bioanalytical Mass Spectrometry Group, Max Planck Institute of Biophysical Chemistry, 37077 Göttingen, Germany; 4 Laboratory of Computational Systems Biology, German Center for Neurodegenerative Diseases (DZNE), 37075 Göttingen, Germany; 5 Center for Biostructural Imaging of Neurodegeneration (BIN), 37075 Göttingen, Germany; 6 Genes and Behavior Department, Max Planck Institute of Biophysical Chemistry, 37073 Göttingen, Germany; 7 Department of Plant Biochemistry, Albrecht-von-Haller-Institute, Georg-August-University, 37073 Göttingen, Germany; 8 Department of Cellular Logistics, Max Planck Institute for Biophysical Chemistry, 37073 Göttingen, Germany; 9 Laboratory of Epigenetics in Neurodegenerative Diseases, German Center for Neurodegenerative Diseases (DZNE), 37075 Göttingen, Germany; 10 Department of Psychiatry and Psychotherapy, University Medical Center Göttingen, 37075 Göttingen, Germany; 11 Department of Cellular Biochemistry, University Medical Center Göttingen, Göttingen, 37073 Germany; 12 Max Planck Institute for Biophysical Chemistry, 37073 Göttingen, Germany; 13 Institute of Medical Systems Biology, Center for Molecular Neurobiology (ZMNH), University Medical Center Hamburg-Eppendorf (UKE), 20246 Hamburg, Germany; 14 German Center for Neurodegenerative Diseases (DZNE), 72076 Tübingen, Germany

## Abstract

The homeostasis of the proteome depends on the tight regulation of the mRNA and protein abundances, of the translation rates, and of the protein lifetimes. Results from several studies on prokaryotes or eukaryotic cell cultures have suggested that protein homeostasis is connected to, and perhaps regulated by, the protein and the codon sequences. However, this has been little investigated for mammals *in vivo*. Moreover, the link between the coding sequences and one critical parameter, the protein lifetime, has remained largely unexplored, both *in vivo* and *in vitro*. We tested this in the mouse brain, and found that the percentages of amino acids and codons in the sequences could predict all of the homeostasis parameters with a precision approaching experimental measurements. A key predictive element was the wobble nucleotide. G-/C-ending codons correlated with higher protein lifetimes, protein abundances, mRNA abundances and translation rates than A-/U-ending codons. Modifying the proportions of G-/C-ending codons could tune these parameters in cell cultures, in a proof-of-principle experiment. We suggest that the coding sequences are strongly linked to protein homeostasis *in vivo*, albeit it still remains to be determined whether this relation is causal in nature.

## Introduction

Proteins are crucial mediators of cellular processes, and the regulatory mechanisms that ensure the proteome homeostasis are essential for living organisms. Proteome homeostasis is a dynamic equilibrium that relies on several events, including the regulation of protein synthesis, abundance, folding, trafficking, sub-cellular localization and degradation^1^. The proteome homeostasis is also intimately linked to the mRNA homeostasis, although the variation in mRNA levels alone is not sufficient to explain the variation in protein abundance^2^. Proteome regulation is an exceedingly complex process^3^, with many levels ranging from genomic architecture and chromatin packing^4,5^ to mRNA modifications^6^, protein modifications, or protein degradation^1^. This complexity is required not only for maintaining cellular function, but also as a response to challenges as the imminent threat of a pathogen, which leads to the production of large new sets of proteins^7^.

It is thought that the various regulatory processes behind protein homeostasis are coordinated by a common framework, the nature of which has remained a fundamental challenge in biology^8^. Emerging studies indicate that such a framework might be encoded directly in the codon and amino acid sequences^9^. In fact, several correlations have been observed between sequence features and parameters of protein homeostasis, such as mRNA and protein levels, translation rates and protein lifetimes^10–17^. In addition, other elements that are known to affect the protein life cycle, as the localization to specific organelles and/or the incorporation in specific protein complexes^18^ may be encoded in the sequences, but in a fashion that is yet to be fully defined.

The literature on the correlation between the protein homeostasis and protein or codon sequences has mainly been concerned with single-cell organisms, where precise quantifications are easier to obtain. As an example, in *E. coli* careful protein and mRNA measurements could demonstrate that the codon content is able to control both mRNA folding and protein translation^19^. Substantial work has also been performed in the yeast *S. cerevisiae*, which has been one of the first cellular systems where prediction models for the global regulation of the proteome have been developed^20,21^. Such models enabled the estimation of protein abundances from mRNA expression data, taking into consideration tRNA adaptation indexes and evolutionary rates. More recently, as an extension of these models, protein and codon sequences have been implemented to predict mRNA levels, translation rates, and protein levels^22,23^. In this context, the correlations with protein levels were highest for the sequences of the open reading frames of mRNAs, suggesting that the information in the untraslated regions (UTRs) is largely redundant for the prediction protein stability. These findings are complicated by the recent discovery of a 3′ UTR motif that explains most of the variability of mRNA levels in yeast^24^.

Such works have also been performed in mammalian, and in *Drosophila* cell culture^25,26^. For example, the degradation of mammalian mRNAs has long been correlated to the presence of AU-rich mRNA motifs^27^, and protein expression regulation has been correlated to the motifs contained in the ORF regions of mRNAs^26^. The connections between mammalian sequences and parameters of the protein homeostasis have been suggested to be causal in nature, as indicated by protein expression experiments relying on different synonymous codons, which suggested that the codon bias^14^, the mRNA secondary structure^28^, and/or the G/C contents^29^ may induce changes in protein homeostasis parameters, and/or in the conformation of individual proteins^30,31^. However, no complete consensus exists on the causality issue, and the optimal interpretation of such experiments is still unclear^32^.

In contrast to the abundant information on prokaryotes and eukaryotic cell cultures, the connections between the protein sequences and protein homeostasis have been less explored for multicellular organisms, and especially for mammals *in vivo* (albeit several works have already focused on simpler multicellular organisms such as the bread mold *Neurospora crassa*^33,34^). In addition, an important parameter of protein homeostasis, the protein lifetime, has been only tangentially connected to sequence features^35^, and, to our knowledge, has not yet been considered in relation to codon proportions, neither in single-cell organisms, nor in mammalian cell cultures or living mammals. We therefore decided to test the link between the amino acid and codon sequences and the parameters of proteome homeostasis, focusing on the mouse brain. This choice was due to the following arguments. First, neurons, one of the main cell types in the brain, are long-lived cells with extremely limited self-renewal capacity. Thus, they require an optimal control of homeostasis to prevent the accumulation of misassembled proteins, or the insufficient production of necessary proteins. As such the brain has a very limited regenerative capacity and will depend more strongly on accurate protein homeostasis than most other organs. Second, the brain is a highly safeguarded tissue, and is maintained in virtually unchanging conditions. Therefore, its proteome would be considerably less variable over time than, for example, that of the liver or of the muscle, which respond to different conditions (*e.g*. feeding regime or activity regime) much more strongly than the brain, rendering the brain proteome more easily predictable. Finally, extensive *in vivo* and *ex vivo* measurements are feasible in the brain, for parameters such as protein and mRNA abundances, protein lifetimes, and translation rates. We performed such measurements, and we found that the protein lifetime, along with other parameters of homeostasis, could be predicted with fairly high precision from the sequences, with the most important sequence parameter being the G/C contents of the third (wobble) nucleotide.

## Results

### Different protein homeostasis parameters show similarities across different tissues and conditions

We set out to test whether the amino acid and the codon sequences could predict the different parameters of protein homeostasis in mammals, such as the protein lifetimes, the mRNA and protein abundances, and the translation rates. Before proceeding to this work, we needed to test the context-dependent variability of these parameters among different tissues and conditions, to make sure that it is possible (and reasonable) to search for connections between the sequences, which are the same for all tissues, and the homeostasis parameters, which may vary substantially among different tissues and conditions. As mentioned in the Introduction, we expect tissues to respond to different conditions by changes in their cell populations and proteomes. The essential question in this context is whether such changes are extensive or whether they are relatively limited.

For this purpose, we first considered the protein abundances, since here we can rely on many previously published works that have produced high-precision data. Based on an extensive study of protein abundances^36^, we found that these show similarities across different tissues in human (Supplementary Fig. 1a). As discussed in the original study, individual protein species do change in abundance in particular tissues. For example, as expected synaptic proteins are more abundant in the brain than in all other tissues. However, despite the within-gene differences that occur between tissues, the relative abundances of the proteins are overall strikingly similar, when regarded at the level of the whole proteome (Supplementary Fig. 1b), with highly significant correlation coefficients (*r*^2^) that are on average > 0.5 for most tissue comparisons. This is not an often-discussed result in the literature, where most observations understandably focus on the differences between tissues and conditions. Nevertheless, this similarity for the protein abundances in different tissues and conditions is confirmed in other extensive studies^37,38^. These similarities have also been observed for mRNA abundances^36,37,39–43^ translation rates^44,45^ and protein lifetimes^18,41,46^; Supplementary Fig. 1c). Along the same lines, a recent work that has analyzed the variables that can predict protein lifetimes *in vivo* has revealed that protein lifetimes measured *in vitro* hold the highest predicting power^47^, suggesting that for some homeostasis parameters there are intrinsic similarities that are conserved both *in vitro* and *in vivo*.

Overall these findings reinforce the idea that there might be some common mechanisms among tissues and conditions, at least under steady-state/physiological situations, that regulate different homeostasis parameter. What the exact biological meaning of these similarities is surpasses the scope of this work. At the same time, these results do not exclude that there are differences among tissues, which might be regulated by sequence-related processes, such as codon optimality^48,49^. We could summarize these apparently contrasting concepts as follows: both specific and common regulatory mechanisms coexist in tissues to regulate their homeostasis. Common mechanisms (common to all tissues) are needed to regulate the housekeeping pathways on which cells depend for their survival, and which are similar among all cell and tissue types. The tissue-specific mechanisms are essential for the terminal differentiation and the specific function of each tissue.

We therefore conclude that the protein and mRNA abundances, the translation rates, and the protein lifetimes are similar enough among the tissues of an organism (and even among related organisms; see examples in Supplementary Fig. 1d) to test whether they can be predicted from a number of variables including codon and protein sequence composition.

### The protein lifetimes correlate to amino acid and codon abundances

To engage with the predictions, we first turned to the protein lifetimes, which have been the least studied in this context, as discussed in the Introduction. We performed all experimental work on the mouse brain, for the reasons mentioned in the Introduction. We initially performed all predictions on our own experimental data, but we always compared our results with the literature, as described below for each measurement and prediction.

**Figure 1:**
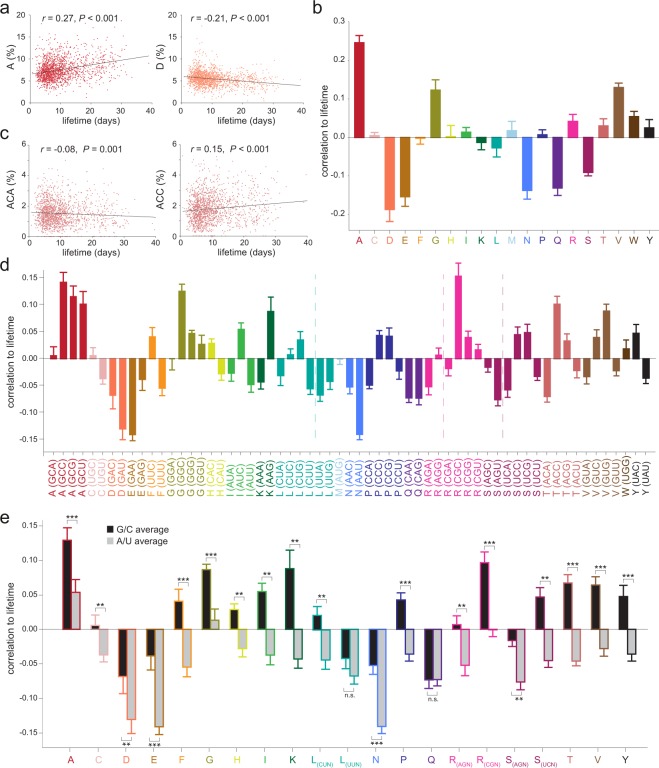
Protein lifetimes correlate to the sequence composition. (**a**) The graphs plot the protein lifetimes against the percentages of alanine (left) or aspartate (right) in the sequences. The alanine (A) percentage correlates positively with the lifetime, while the percentage of aspartate (D) correlates negatively. The two amino acids also anticorrelate significantly with each other (*r* = −0.28; *P* < 0.001). (**b**) Pearson’s correlation coefficients of the percentages of amino acids against the protein lifetimes. (**c**) The graphs plot the protein lifetimes against the percentages of two synonymous codons (ACA and ACC). Despite coding for the same amino acid (threonine), these codons are differently correlated to protein lifetimes, and anticorrelate significantly with each other (*r* = −0.11; *P* < 0.001). (**d**) Pearson’s correlation coefficients of the percentages of single codons in the mRNAs against the protein lifetimes. We plot this value according to the encoded amino acids, and color-coded as in panel b. Three amino acids (leucine, arginine and serine) are encoded by 6 codons, from two different subgroups: a pair with identical nucleotides at the first two positions, and a quadruplet, again with identical nucleotides at the first two positions. For clarity, the pairs and quadruplets are separated with segmented lines. The differences between codons of individual amino acids are not random: codons ending with a C or a G tend to have more positive correlation values than codons ending in A or U. This tendency holds true for 20 out of 21 codon subgroups, the only exception being the glutamine-encoding codons (CAA and CAG, purple). (**e**) Summary of the correlations expressed as averages for the G-/C- or A-/U-ending codons. All differences are significant, with the exception of the UUN codon group (leucine) and of the glutamine codon group. Student’s t-test significance levels within the same codon group: **P < 0.01, ***P < 0.001. Error bars = s.e.m. For panels b, d and e, *n* = eight datasets from 4 independent lifetime studies^18,41,46,50^.

We defined the protein lifetimes as the average time period spent between protein production and degradation. Along with our own dataset of protein lifetimes in the mouse brain^41^, we also relied on several sets of protein lifetimes obtained in the mouse brain, and, for comparison purposes, in the mouse heart and in primary neuronal cell cultures^46,50,51^. We first tested whether the protein lifetimes were related in any fashion to the amino acid composition of the proteins. We calculated the amino acid composition of all protein sequences in our database, in percentages. For example, the % of alanine in the proteins in our database spans from ~2% to ~20% (~5–10% for most proteins). We then plotted the percentages, for each amino acid, against the lifetimes of the respective proteins (see examples in Fig. 1a). To obtain a numeric value representative for this plot, we calculated the Pearson correlation coefficient (*r*) between the amino acid percentages and the protein lifetimes (Fig. 1b). Three hydrophobic and/or non-polar amino acids, alanine (A), glycine (G) and valine (V), correlated positively to the lifetimes (Fig. 1a,b). The opposite was observed for five negatively charged or polar amino acids, aspartate (D), glutamate (E), asparagine (N), glutamine (Q) and serine (S, Fig. 1a,b).

We then performed the same type of measurement for the codon sequences (see examples in Fig. 1c). To our surprise, we found that synonymous codons had different behaviors, and that, overall, the codons ending with a cytosine (C) or guanine (G) were more positively correlated with the protein lifetimes than codons ending in adenine (A) or uracil (U, Fig. 1d). This was true for all of the synonymous codon groups, with the sole exception of the two glutamine codons (CAA and CAG). Moreover, for 12 amino acids the C- and G-ending codons correlated positively to protein lifetimes, while the synonymous A- and U-ending codons correlated negatively. This is easily visualized if the coefficients of the G- and C-ending codons, and of the A- and U-ending codons, are averaged for each synonymous codon group (Fig. 1e).

### The protein lifetimes can be predicted from the amino acid and codon sequences

**Figure 2:**
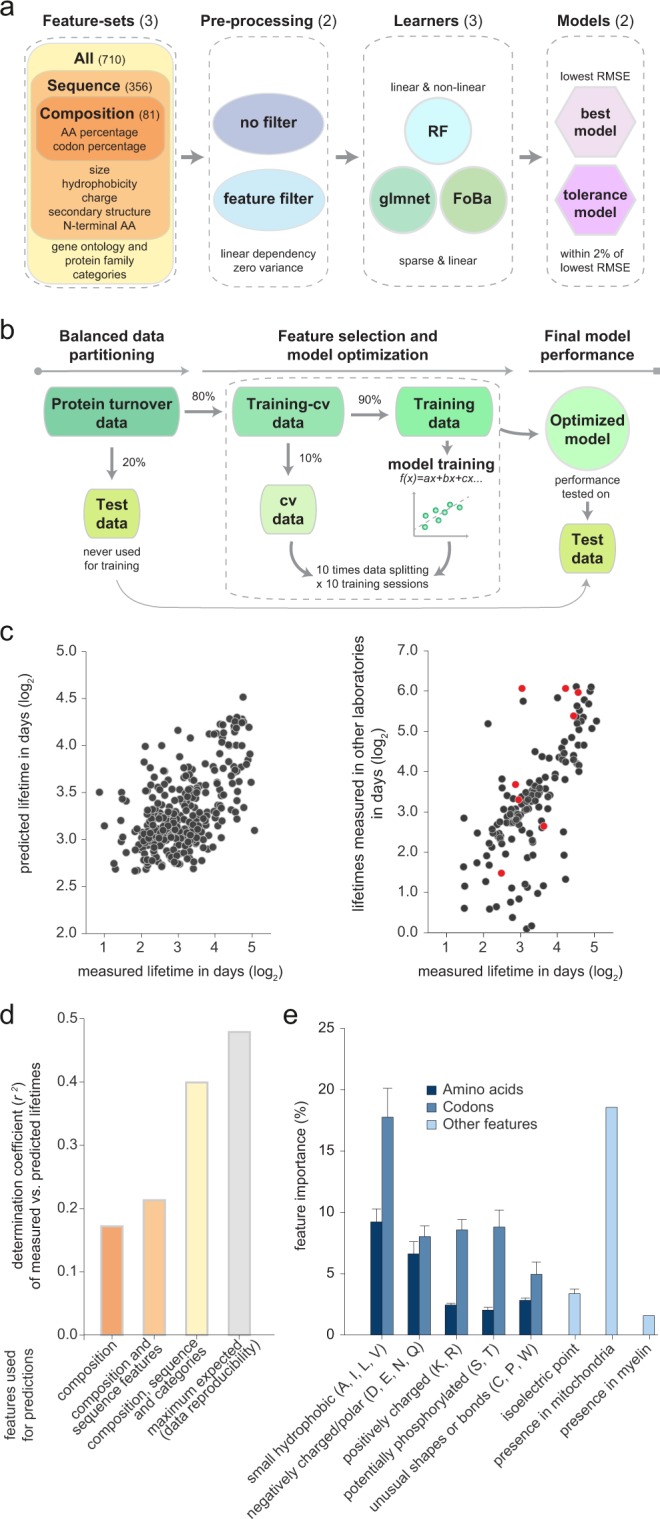
The amino acid and codon sequences can be used to reliably predict protein lifetimes. (**a**) Overview of the 36 models used for predicting lifetimes. Models were learned for three feature sets: (1) protein composition features (amino acid and codon percentages; 81 features); (2) composition features and 275 additional features derived from sequences (*e.g*. secondary structure information, length, etc.); (3) all previous features and 354 additional features. Models were tested with or without filtering features that are redundant, using three different learning algorithms (RF, glmnet and FoBa) and reporting the best (lowest RMSE) and the tolerance models. (**b**) Model optimization. The protein turnover dataset is split into test set (20%) and training-cross-validation set (80%). The training-cross-validation set is further split into training set (90%) and cross-validation (cv, 10%). The best cv-model, is obtained using a 10-fold cross-validation with 10 initializations. Performance of each model is evaluated on the test set comparing the RMSE of the predictions to the observed values. (**c**) Left: example scatter plot showing lifetimes measured in this study against respective predicted lifetimes (based on all features). Only the 20% test data relevant for measuring prediction precision are shown. Right: experimental variation between independent samples for this set of protein lifetimes, plotting our lifetimes against the respective values from two other available studies from the literature (black^18^; red^57^). (**d**) Pearson’s correlation coefficients between measured and predicted lifetimes by three random forests (RF) models based on sequence composition alone, on all sequence features, or on the entire feature list. For comparison, correlation coefficient between different published lifetime datasets^18,57^ (maximum expected). (**e**) Most important protein features in predicting the lifetimes, as defined by models. Amino acids were grouped in small hydrophobic (A, I, L, V), negatively charged/polar (D, E, N, Q), positively charged (K, R), potentially phosphorylated (S, T), and unusually shaped/bonded (P, W, C). Sum of the importances of these amino acids, or of their codons, was calculated and plotted. Bars: average importances of the respective features in the three RF models; error bars: s.e.m. Gene ontology features lack error bar since they are present only in the third model.

We therefore concluded that the protein lifetimes correlate to different codon and amino acid percentages, albeit these correlations were relatively weak (*r* ranging from −0.21 to 0.27). To test whether such sets of correlations could predict the protein lifetimes, we used three regression machine-learning approaches to build predictive models (Fig. 2a,b and Supplementary Fig. 2). Based on the evidence that several features are linearly correlated, we choose two linear approaches including linear glmnet^52^ and FoBa regression^53^, and a third “random forests” (RF) approach^54^ that can also capture non-linear dependencies. Datasets were split into training (72%), cross-validation (8%), and test (20%) sets, and models were fit using 10-fold cross-validation (Fig. 2b; see also Methods). Overall, the root mean squared error of the three regression approaches achieved similar cross-validation and test performances, although non-linear relations were appropriately modeled only by the RF approach, which was therefore chosen for building all the models presented in this study (Supplementary Fig. 2h,i). To avoid the risk of overfitting^55^ when discussing the results of the models, we relied only on the test performances (for an example see Fig. 2c).

We used three RF models (Fig. 2a). First, a model including only the sequence composition, with 81 features: 20 amino acid percentages, and 61 codon percentages. Second, a model containing the sequence composition, and several additional sequence-derived features, as follows. We relied on the Psipred^56^ software to separate each sequence in estimated α-helix, β-sheet and random coil regions, and calculated the percentage of each amino acid or codon in these regions. We also added overall sequence features such as the length and the molecular weight, the grand average of hydropathy (GRAVY), the aliphatic index, the nature of the N-terminal amino acids^15^, and the theoretical isoelectric point (as detailed in Methods and in Supplementary Dataset 1). Third, a model containing all of the previous composition and sequence-derived features, together with 354 features from published databases, including gene ontology (cellular component, molecular function, and biological process), and protein family affiliations (PFAM). We defined these as “non-sequence features”, albeit some are derived from the sequences, as the protein family affiliations, the localization to some organelles (via signal peptides), and the molecular function (whose assignment is largely based on domain sequence homologies). Others may be encoded in the sequences, but in a fashion that is yet to be fully defined, such as localization to mitochondria. Yet others may not be encoded in the sequence at all, as for some of the cellular component tags.

The lifetimes predicted by the RF model based on the sequence composition alone (*i.e*., amino acid and codon percentages alone) correlated to the experimentally measured lifetimes significantly (Fig. 2d, *r* = 0.41, *P* < 0.001). Virtually all codon and amino acid percentages were used in the prediction (Supplementary Dataset 1). Higher feature importances were assigned to the percentages of alanine and aspartate, and to those of some of their codons, as expected from the fact that they have the strongest positive (alanine) and negative (aspartate) correlations to the lifetime (Fig. 1b).

While the value of the correlation between the measured and the predicted lifetimes appears small, it should be noted that the maximal possible correlation is similar to the experimental variation between independent samples, since values that cannot be reproduced between measurements are probably erroneous, and are unlikely to be predicted by any computational model. Lifetimes from independent studies of the brain^18,57^ correlated to each other with an average *r* of ~0.69, implying that the *r* obtained from the prediction corresponds to ~60% of the maximum achievable. Importantly, the literature relating protein homeostasis parameters to each other (*e.g*., mRNA amounts to protein amounts or production rates), the coefficient of determination, meaning the squared Pearson correlation coefficient (*r*^2^) has often been used to reflect correlations, rather than the *r* value^58^. We therefore employ here the *r*^2^ when referring to the strength of our predictions (see Supplementary Dataset 2 for a comparison of both *r* and *r*^2^ values for all predictions). When considering the *r*^2^, this model’s prediction (*r*^2^ = 0.17) corresponds to ~35% of the maximum expected value (*r*^2^ = 0.48; Fig. 2d).

Using the second model, containing the sequence composition and other sequence-related features, we predicted lifetimes that resulted in a coefficient of determination of *r*^2^ ~0.21 to the measured values (Fig. 2d), corresponding to ~44% of the maximum expected correlation. The most important features in this prediction were once more the amino acid and codon percentages of alanine and aspartate, along with their presence in random coil (aspartate) or helix (alanine) structures (Fig. 2e). The isoelectric point, the GRAVY, and the overall percentage of negatively charged amino acids were also important features (Fig. 2e and Supplementary Dataset 1).

N-terminal amino acids have been considered essential components of degradation signals, also known as N-degrons, leading to the formulation of the N-end rule^15,59,60^. Interestingly, the nature of the N-terminal amino acid did not result in any improvement of the predictive power, as confirmed by the almost absent influence of the N-terminal amino acid on the protein lifetimes (Supplementary Fig. 3a), and by the inexistent correlation between protein lifetimes measured *in vivo* and the N-end rule (*r*^2^ = 0.02, Supplementary Fig. 3b). Not surprisingly, the same was observed for each single codon independently (Supplementary Fig. 3c,d). The same was also observed for a more extensive list of known degron motifs^61^. The observation that degrons seem not to have an influence on protein lifetimes is counterintuitive, but can be explained by the fact that at the global level other features are more predictive, and the information of degrons might be redundant.

**Figure 3:**
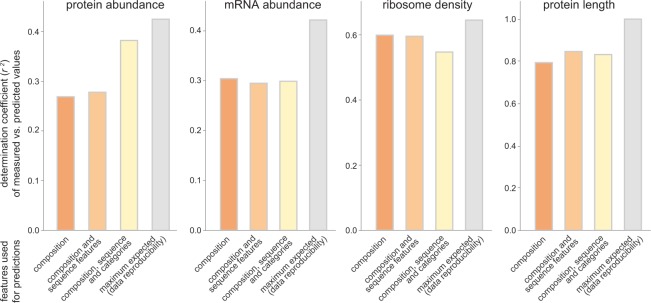
The protein and mRNA abundances, the ribosome density and the protein length can be predicted from the sequences. We used the same models as in Fig. 2 to predict the protein and mRNA abundances, the ribosome density and the protein length. As in Fig. 2, the models were based on (1) the protein composition – the amino acid and codon percentages; (2) these percentages and features derived from the sequence; (3) all of the previous features and additional overall features. The bar graphs show the Pearson’s correlation coefficients between the measured protein and mRNA abundances, ribosome densities and lengths, and the respective values predicted by our models, as in Fig. 2d. Remarkably, the amino acid and codon compositions alone are sufficient to predict these parameters with an accuracy that is on average ~70% of the maximum expected (the reproducibility of the data between different data sets, from different laboratories).

Finally, we employed the model containing all sequence and non-sequence features described above. This increased the correlation to an *r*^2^ ~0.39, or 81% of the maximum expected value (Fig. 2d). This implies that this model can predict protein lifetimes almost as well as they can be measured. Most non-sequence features were actually of limited predictive value. The most important ones were localization to mitochondria, myelin sheath, synaptic vesicle, and to the cytoskeleton (Fig. 2e and Supplementary Dataset 1). This is in line with the observation that these structures have longer lifetimes than most other components of the brain (our observation^41^).

### Other parameters of protein homeostasis can be predicted from the amino acid and codon sequences

Having established that one key parameter of protein homeostasis, the protein lifetime, can be predicted by the sequence composition, we next sought to verify whether this is a general characteristic of homeostasis in mammalian tissues. We first sought to verify this in the same tissue we used for the protein lifetime predictions, the mouse brain. We measured protein abundances using iBAQ^62^, and mRNA abundances by whole transcriptome shotgun sequencing^63^. For the analysis of ribosome density we relied on published Ribo-seq data^64^, although this type of measurement has been under some scrutiny lately^65^. These parameters correlate to each other to varying extents (Supplementary Fig. 4), with *r*^2^ values ranging from 0.003 to only 0.178, in line with the available literature, which indicates that the protein homeostasis parameters are not linked to each other with very high strength^58,66^.

All of these parameters correlated to the amino acid and codon sequences, in the same fashion as the protein lifetimes did (Supplementary Fig. 5), and they all could be predicted from the RF models we introduced above (Fig. 3). As the protein length seems to be connected to homeostasis parameters^16^, we also included it in the predictions. Since this parameter is precisely known, it was important to test whether it can also be predicted with high precision. The amino acid and codon composition predicted the protein abundance, the mRNA abundance, the ribosome density, and the protein length to significant levels, approaching the accuracy with which these parameters can be reproduced between datasets: respectively 90%, 75%, 84% and 83% of the maximum expected coefficients of determination, *r*^2^, which refer to the experimental variation between independent studies. The experimental variation between studies was determined by comparing our dataset with mouse brain protein abundance datasets^38^, and with mouse mRNA abundance datasets^39,64,67,68^, or by calculating the variability between independent measurements in the published Ribo-seq data we used^64^. While the experimental reproducibility of mass spectrometry measurements is becoming excellent^69^, the values reported in our analysis also take into account the reproducibility of the animal housing and animal care conditions, and of the sample preparation steps taken to obtain the proteins, peptides or RNA molecules analyzed in the different laboratories. The length was considered to have no variability, meaning that the maximum expected correlation is 100%. The relative importance of each of the codons and of the amino acids in all of the predictions is detailed in Supplementary Dataset 1.

### These observations apply also to other multicellular organisms

We next investigated whether these observations made in mouse would translate to other organisms. Lifetimes *in vivo* have not yet been extensively studied in vertebrates other than the mouse, so we cannot state whether the same sequence correlations apply to, for example, human tissues. However, the other parameters (protein and mRNA abundances, ribosome densities and the protein length) have been studied in numerous model organisms. Using 800 published data sets, representing different tissues and conditions, from 25 different studies (see Supplementary Dataset 3 for the additional references), ranging from *E. coli* to human, we determined that the codon correlations to the different parameters are very similar among mammals (mouse, rat and human), mammalian (human) cell cultures, and for zebrafish (Supplementary Fig. 6). Furthermore, RF models built with murine data were able to predict the human protein abundance, mRNA abundance, ribosome density and protein length (with *r* values on average only 18% lower than when predicting the respective mouse parameters). Conversely, the human data could be used to predict the mouse parameters (with almost identical performance to predicting the human parameters). The correlations are far poorer for invertebrates and plants, and even poorer for unicellular organisms, suggesting that the correlations described here apply primarily to vertebrates and more specifically to mammals.

### A proof-of-principle experiment suggests that the protein lifetimes, along with the other parameters of homeostasis investigated here, can be tuned by altering the codon proportions in the sequences

Having thus verified that the amino acid and codon sequences can predict various cell biology parameters, we sought to provide a proof-of-principle experiment that would determine whether a manipulation of the sequences could change such parameters. This remains a hypothetical question for the amino acid sequence, since any modification would change the protein too drastically for meaningful interpretation. However, this question can be approached from the codon perspective, since the codon composition effects were not random, but depended on the G/C versus A/U nature of the wobble nucleotide. This nucleotide can be manipulated without changing the amino acid sequence, which opens the possibility of testing whether the sequence composition influences these parameters. This has already been suggested, for example, for the translation rate in bacteria^70,71^, or for the protein and mRNA amounts in HeLa cells^29^. For this purpose, we initially generated five synthetic genes based on the wild-type sequence of α-synuclein (a protein whose pathogenic accumulation has long been connected to Parkinson’s disease^72^) linked to a SNAP-tag^73^, which enables pulse-chase experiments. All five genes coded for the same amino acid sequence, but contained different percentages of G-/C-ending codons, ranging from 0% to 100% (Supplementary Dataset 4). The resulting mRNAs had, as expected, different folding strengths, with the G-/C-containing ones being most strongly folded (with an estimated ΔG decreasing linearly from −220 to −420 kcal/mol with increasing percentages of G-/C-ending codons).

**Figure 4:**
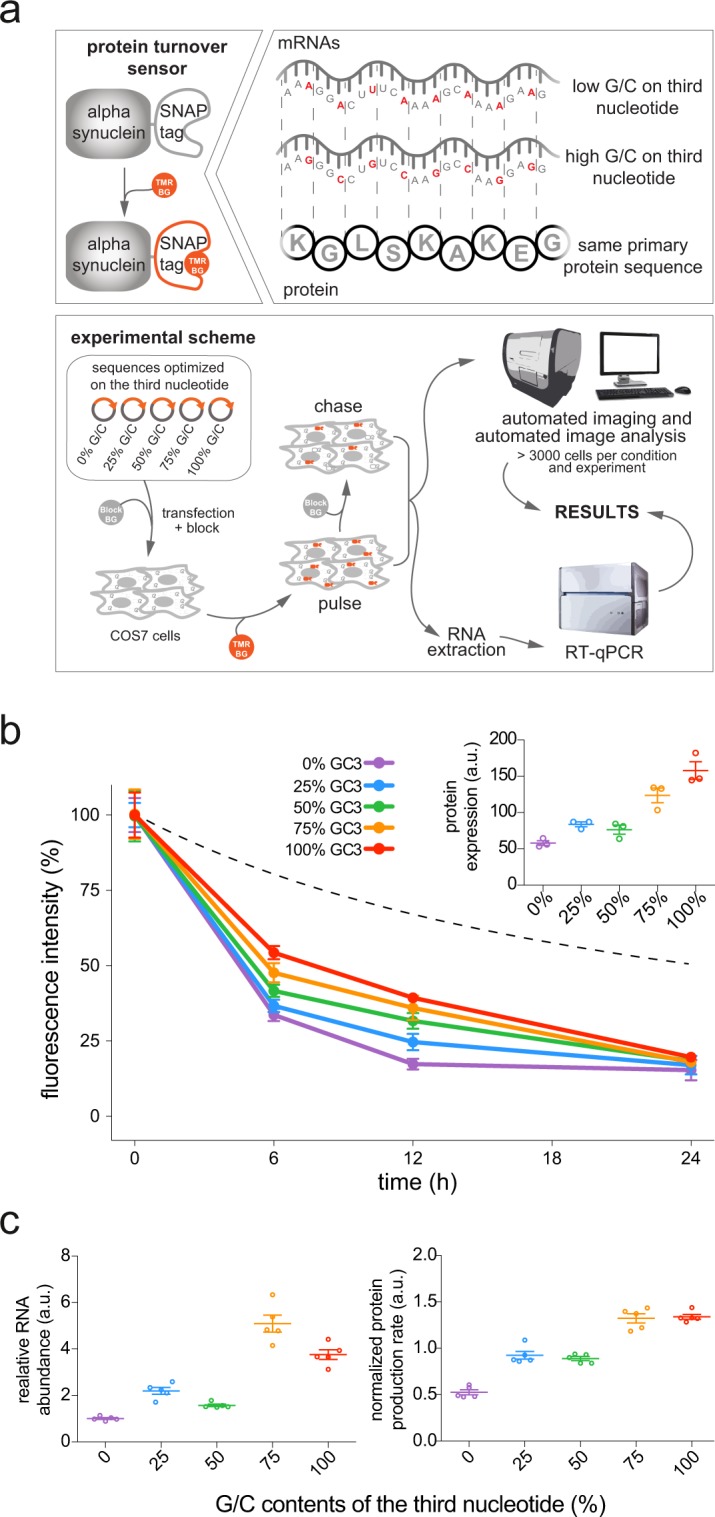
The nature of the third nucleotide coordinates a number of parameters linked to protein homeostasis. (**a**) Scheme of the approach. The turnover sensor is composed of the wild-type sequence of α-synuclein (a protein connected to Parkinson’s disease) fused to SNAP-tag^73^. This sensor is covalently labeled with an O^6^-benzylguanine tag labeled with tetramethylrhodamine (TMR-BG), revealing the turnover of proteins in pulse-chase experiments. We optimized five versions of this sensor with increasing percentages of G-/C-ending codons (0%, 25%, 50%, 75% and 100%). The five plasmids were transfected in equimolar quantities. Before the pulse, pre-existing sensors were blocked, to label exclusively the newly synthesized proteins, using an unlabeled tag (BLOCK-BG). Cells were then pulsed for 2 h with TMR-BG and chased in BLOCK-BG, to reveal turnover. Following the chase, cells were fixed, imaged and analyzed in an automated fashion. In parallel, mRNA samples were collected, retrotranscribed, and mRNA levels were analyzed by RT-qPCR. The LightCycler 480 (Roche Life Science) and the Cytation 3 Cell Imaging Reader (BioTek) were used for these experiments and the creation of these scheme. (**b**) Results of the pulse chase experiment, showing decay of the labeled sensors encoded with increasing percentages of G-/C-ending codons. Segmented line: percentage of the dye lost by cell division dilution. Protein lifetimes are increased for sequences with higher percentages of G-/C-ending codons, as shown by slower decay of the sensors with higher G-/C-ending codons (see also Fig. 5f). Each dot represents the average of three separate experiments with SEM (*n* = 3). Similarly, higher percentages of G-/C-ending codons increase protein abundance, as shown in the inset that represents the relative protein expression at the beginning of the experiment (time_0_), measured as absolute fluorescence for each sensor. (**c**) Turnover parameters analyzed during the experiment comprising the mRNA abundance (left), and the protein production rate (right). Each dot represents a separate experiment (*n* = 5). Not only the protein lifetime is increased for sequences with higher percentages of G-/C-ending codons, but also mRNA abundance and protein production rate. The graphs show means from 5 independent experiments. Trends statistically significant for all graphs, linear regression *P* < 0.001.

These genes were transiently transfected in a mammalian fibroblast cell line (COS7), and the cells were pulsed with an O^6^-benzylguanine (BG) derivative carrying a modified rhodamine dye, which becomes covalently bound to the SNAP-tag (Fig. 4a). This was followed for 24 h in a chase experiment, to monitor the degradation of the fluorescently-pulsed proteins. In parallel, the mRNA amounts were monitored by reverse transcription polymerase chain reaction (RT-qPCR)^74^. The results confirmed that the codon composition determines the turnover parameters. Cells transfected with constructs containing higher percentages of G-/C-ending codons had the strongest fluorescence intensity, and lost the fluorescence more slowly (Fig. 4b), indicating that both the protein lifetime and the protein abundance were larger (Fig. 4c). The same was observed for the mRNA abundance and the protein production rate (Fig. 4c).

**Figure 5:**
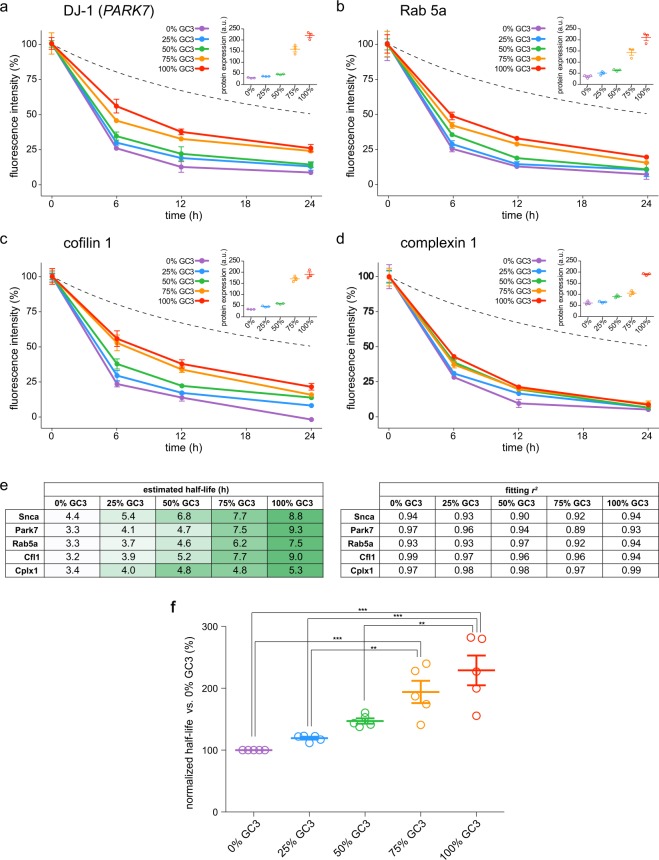
The nature of the third nucleotide influences protein lifetimes. (**a–d**) We designed four additional families of protein turnover sensors composed of the sequence of four different proteins fused to the SNAP-tag. For each one of the four protein species, we optimized five versions of their coding sequences with increasing percentages of G-/C-ending codons (0%, 25%, 50%, 75% and 100%), corresponding to 20 additional constructs. For each protein, the five plasmids with increasing percentages of G-/C-ending codons were separately transfected in COS7 cells in equimolar quantities. Also in this case, as for Fig. 4b, the sensors were pulsed for 2 h and chased for 24 h, revealing the turnover of the different proteins. For each desired chase time, cells were fixed, imaged with a high content microscope and the fluorescence analyzed in an automated fashion. The segmented lines represent the percentage of the dye that was lost due to the dilution caused by cell division from the beginning of the experiment. Each dot in the graphs represents the average of three separate experiments with SEM (*n* = 3). The inset in the upper right corner of each graph shows the relative protein expression at the beginning of the experiment (time_0_), measured as absolute fluorescence of every sensor. Each panel shows the results from >10.000 cells analyzed. (**e**) Summary of the lifetimes (expressed as t_1/2_), calculated through the fitting of the data represented in in Fig. 4b for Synuclein (Snca) and in panels a–d for the remaining proteins. The *r*^2^ indicates the coefficient of determination for each fitting used for the calculation of the lifetimes. (**f**) Lifetimes increase *versus* the 0% GC3 condition. Higher G-/C-ending codon percentages continuously increase the t_1/2_ from 0% to 100% GC3 (statistically significant trend with a linear regression *P* < 0.0001). With respect to the 0% GC3, in the 100% GC3 the lifetimes of these sensors are increased on average up to 229.0 ± 24%, roughly doubling their stability. ANOVA followed by Bonferroni post-hoc comparisons test: **P < 0.01, ***P < 0.001. Error bars = s.e.m.

To confirm that the effects of the G/C-ending codons on protein lifetimes were not specific for alpha synuclein, which might undergo complex oligomerization events^75^, we extended the protein turnover experiments to four additional genes (complexin-1, cofilin-1, protein DJ-1/Park7 and Rab5a), for a total of 20 other sequences with GC contents ranging from 0% to 100% (Supplementary Dataset 4). Since the measurements of protein stability might be influenced by our detection system, we performed a control calibration of all our imaging settings, which allowed us to rely on the precision of our measurements (Supplementary Fig. 7). We also checked that the lifetimes that we measured are compatible with lifetimes measured with analogous imaging approaches^76^ (Supplementary Fig. 8). Altogether, these experiments reveal that, although variably, the effects of G/C-ending codons on protein turnover and protein abundance can be extended to different protein species (Fig. 5a–d). In detail, higher G-/C-ending codon percentages increasingly extend the lifetimes of these sensors, roughly doubling their stability when comparing the lifetimes of 0% to 100% G/C-ending sensors (Fig. 5e,f).

This implies that changing the composition of the synonymous codons can tune all of the parameters of protein homeostasis measured here (although such *in vitro* experiments have different levels of uncertainty that cannot be controlled perfectly, including, for example, the use of codons that may be particularly under-represented in the tRNA population of the cell line utilized).

## Discussion

In this work we tested the correlation between protein and codon sequences, alongside with other features, and several basic parameters of protein homeostasis obtained from *in vivo* data, in the mouse brain. We provide an extensive characterization integrating more than 800 multi-omics data sets, from several published studies. Our results demonstrate that both codon and amino acid sequences hold a predictive power in the determination of protein lifetimes, protein abundances, mRNA abundances and translation rates in the mouse brain. The data also imply that the wobble nucleotide could be used to fine-tune multiple protein turnover parameters, which should prove interesting for synthetic biology and protein production technologies, in agreement with previous work on this issue^29^.

### The predictability of proteome parameters

The idea of a protein and codon sequence-based regulation of the proteome *in vivo* might raise substantial skepticism. This is in part due to the fact that there is a disproportioned amount of experimental work that deals either with tissue-specific differences, or with dynamic changes of the proteome following a perturbation (such as a stimulus, a toxic insult, the ablation of a protein, or the transition into a new developmental step). Thus, the impression arises that the proteome is continuously changing, and should therefore not be predictable from an unchanging parameter such as the protein sequence. In spite of this impression, the parameters of the proteome are strongly conserved under normal conditions in adult organisms, across different tissues, between different physiological conditions, and even between related organisms (Supplementary Fig. 1). This finding is to some extent expected, given the high similarity of the basic metabolic pathways that are shared by all animal cells, which would leave their proteomes broadly similar, and therefore potentially predictable. At the same time, the work performed here has dealt with the brain, an organ which is expected to change little over different conditions. Results in organs that change more extensively over time (liver, muscle, fat tissue, and others) may be somewhat different, and may require additional analyses for accurate predictions.

### The influence of the sequence on protein lifetimes

To our knowledge, this parameter has not yet been considered in relation to the composition of the codon sequence. It has also been just incidentally linked to the amino acid sequence, beyond the nature of the N-terminal amino acid or the protein size and isoelectric point^16,17^. We found that the percentages of some amino non-polar or hydrophobic acids correlated positively to the lifetimes (Ala, Gly and Val), while others, mainly negatively charged or polar, correlated negatively (Asp, Glu, Asn, Gln). These observations could be interpreted by taking into account the possible role of negatively charged residues for increasing protein solubility^77,78^. This would render negatively charged proteins more exposed to damage (*e.g*. oxidative) and to recognition by elements of the degradation pathways. In contrast, hydrophobic proteins may be more tightly packed, or may be buried in membranes, and should therefore receive less damage, and be less easily degraded.

Further effects were seen at the level of the codon sequences, as synonymous codons ending with a C or G were more positively correlated with the protein lifetimes than codons ending in A or U. This correlation is true for all of the synonymous codon groups, with a sole exception (CAA vs. CAG, the Gln codons). This is in line with the suggestion that the nature of the third nucleotide may serve to regulate aspects that could be linked to the stability of proteins, such as protein folding (Hanson & Coller, 2018). However, the observation that the codon percentages tended to be more important in the predictions than the amino acid percentages was surprising, since the former have not been directly linked to protein lifetimes, as indicated above. Many protein features have been used to model protein degradation, with varying degrees of success (for example see^35^), but not the codons. One could argue that, since codons have been linked to parameters spanning from mRNA abundances to translation rates, and since these parameters are related to the protein lifetimes (Supplementary Fig. 4), the lifetimes should also be connected to the codon percentages, thus making our observation expected, and somewhat trivial. However, this is not the case, in view of the relatively poor correlations between the different protein homeostasis parameters in the mouse brain (Supplementary Fig. 4). As often discussed in the literature, these parameters (mRNA and protein abundances, translation rates, protein lifetimes) do not always predict each other with high precision^2^, and thus having one of them predicted by the sequences does not automatically imply that others should be predicted by the sequences as well. This is all the more evident in the fact that different codons were important in the predictions of the different parameters (Supplementary Dataset 1).

From our data it is also apparent that the predictions are more precise when other features are included, such as organelle localization. Hence, the influence of the sequence is not absolute, and non-sequence features are presumably overlaid onto the sequence-derived framework to provide the final values for turnover parameters. Several other features could be investigated, and could be added in the future to improved predictive models. These might include the genomic organization and chromosome architecture^79,80^, the composition and regulation of promoter and enhancer regions^81–83^, the composition of pre-mRNA splicing regions^84^ and of mRNA untranslated regions^85^. At the same time, both yeast and cell culture work indicate that coding sequences are more relevant for the prediction of protein parameters in general than accessory untranslated or regulatory sequences^22,26^.

### The role of degrons and of the N-end rule

Protein sequences contain several degradation motifs, termed degrons. These include the N-terminal amino acids (N-degrons)^15,59,60^. Other specific degrons within each protein sequence are also thought to contribute to protein turnover^61^.

In our models the degrons, including the N-degrons, were features of negligible importance for the prediction of protein lifetimes. This is in line with a recent study that also found little support for the N-end rule in a computational analysis of protein degradation rates in cell cultures^35^. This does not argue against the importance of degrons, or against the N-end rule. Degrons might be implicit in other more general sequence features, which render degron signals redundant in our models, and lead our models to give them a limited importance. We cannot exclude other reasons as, for example, the lack of information about post-translational modifications that are particularly relevant for triggering degradation pathways (as N- terminal acetylation and poly/mono-ubiquitination). Additionally, while it is known that virtually all proteins are translated with a N-terminal methionine, the co-translational cleavage by the methionine N-terminal aminopeptidases is a rather complex process, and the final product of this enzyme is difficult to predict^86–88^. As a technical note, in the original paper establishing the lifetimes which led to the formulation of the N-end rule, the lifetimes were measured from an exogenous protein containing different amino acids at its N-terminus in reticulocytes *in vitro*^59^, an experiment which is linked to the endogenous lifetimes in a limited fashion, although this rule is still used as a gold standard for the prediction of protein half-life in mammalian cells^89^. Our study, which shows stronger predictive power, might serve as foundation for more efficient tools allowing the prediction of protein turnover *in vivo*.

### Several other aspects of the proteome homeostasis are influenced by the sequence

We extended our models to other crucial aspects of the proteome homeostasis, as the protein abundances, the mRNA abundances, and the translation rates. For all models the sequence composition was dominating the predictions, reinforcing the idea that common regulatory mechanisms might take advantage of the unequal use of synonymous codons. Although the discussion of the individual effects of all features is beyond the scope of this work, our list of predictive features, with their relative weights, may serve as a basis for future research and for the fine-tuning of protein production technologies.

The interdependence of the different protein homeostasis datasets could in principle explain why protein lifetimes can be predicted just on the basis of these correlations. Nevertheless, this is not the case for three main reasons: (1) The correlations of other values with the homeostasis parameters are overall low (Supplementary Fig. 4); (2) As previously mentioned the relationships between different codons and the homeostasis parameters are not the same for the different protein homeostasis parameters (Supplementary Fig. 5), reinforcing the concept that the interdependence of these parameters is not high enough to allow cross-predictions and (3) The random forest algorithm that we have used allows to measure the relative importance of each feature in the prediction (as indicated in Supplementary Dataset 1). The relative importances (the weights) of these features in the predictions are different, and sometimes have opposite signs, thus indicating that there is no direct interdependence between different homeostasis parameters. Overall, this indicates that the parameters of protein homeostasis are not enough correlated to efficiently predict protein halftimes.

Intriguingly, the models did not rely on uniquely specific features, such as individual low-abundance codons. In contrast, virtually all amino acids and codons were important in the predictions, implying that the predictive power is distributed along the entire sequence. Importantly, the codon percentages tended to be more important in the predictions than the amino acid percentages, underlining the observation that synonymous codons of one amino acid correlate differently to the various parameters. This is all the more evident for the amino acids with 6 synonymous codons (serine, arginine, leucine), whose codon importances are disproportionally large, implying that the multiple synonymous codons of these amino acids serve the purpose of modulating cellular parameters.

As a side note, in our study we used protein length as a control, since to our knowledge there is no context-dependent regulation of protein length, and since this parameter is very precisely determined for mouse proteins. At the same time, the idea that the sequence composition can predict protein length might be interesting for the discovery of new proteins and for the mammalian proteomes that are yet not studied in detail. The strong correlations seen between all mammals investigated (Supplementary Fig. 6) suggest that such aspects should be conserved also for such proteomes.

Whether the links between the sequence and the homeostasis parameters are causal in nature remains an open question. We do provide a proof-of-principle experiment to test this relationship in cells. One caveat still remains, since GC-rich constructs (which are expressed to a higher level than the low-GC variants) might overload the degradation machinery and thus become more stable. However, while being far from perfect, our approach is based on several experiments, each performed on several thousands of cells, and provides initial evidence for the idea that the parameters of protein lifetimes can be regulated through codon choices. This is in line with previous experiments that have targeted similar genes (albeit not codifying for identical proteins) with 2–4 different G-/C-ending codon percentages^29^, and have studied the resulting protein and mRNA amounts. This issue, however, remains controversial, and awaits further proof, especially from *in vivo* studies.

### The rationale of a mechanism linking codons to protein homeostasis parameters

**Figure 6:**
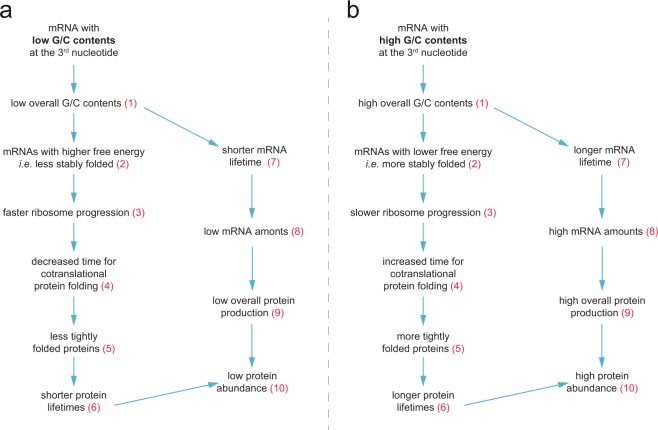
Hypothetical scenarios linking the G/C contents at the third position of codons to protein lifetimes and to other turnover parameters. (**a**) Hypothetical scenario for low G/C contents at the third nucleotide. (**b**) Same scenario for high G/C contents. For simplicity we discuss in detail only the scenario represented on panel a. The same relations apply to the scenario on panel b but in reversed fashion. Molecules of mRNA with a low G/C contents on the third nucleotide will have low overall G/C contents (inference 1; demonstrated in Supplementary Fig. 9a,b). Low G/C contents at the third nucleotide correspond to less stably folded mRNA molecules (inference 2; demonstrated in Supplementary Fig. 9c for mouse brain mRNAs, and Supplementary Fig. 9d for the synuclein synthetic genes). Less stably folded mRNA molecules allow faster ribosome progression (inference 3; reviewed in^10^). Fast ribosome progression decreases the time for co-translational protein folding (inference 4; discussed by Faure and collaborators^90^). This implies that the low G/C contents at the third nucleotide results in less structured proteins (inference 5; demonstrated in Supplementary Fig. 9g). Less structured proteins, *i.e*. with poor folding or with a relatively higher amount of exposed surface, should have shorter lifetimes, since they have larger surfaces that can be affected by damaging interactions, such as oxidation (inference 6; reviewed in^113^, and demonstrated in our previously published results^41^). Low overall G-/C-ending codons decrease mRNA lifetime and result in less abundant mRNAs (inference 7), albeit the precise mechanism is not yet clear for mammals^114^ (inference 8; suggested, with significant albeit not very strong correlations, by our mouse brain data, Supplementary Fig. 9e, and by our data on synthetic genes, Supplementary Fig. 9f). Lower amounts of mRNA then lead to lower protein production rates (inference 9; reviewed by Maier and collaborators^115^). In conclusion, the low G/C contents at the third nucleotide induces shorter protein lifetimes (inference 6) and lower protein production rates (inference 9), which converge to low abundance for the respective proteins (inference 10).

The exact mechanism(s) by which unequal use of synonymous codons can be used to control proteome parameters, especially protein turnover, are still to be understood, especially *in vivo*. We summarize the possible mechanisms in Fig. 6, with some additional supporting information included in Supplementary Fig. 9. One could speculate that a high percentage of G-/C-ending codons lead to mRNAs with a stronger folding energy (Supplementary Fig. 9a–d), due to higher proportion of triple hydrogen GC bonds that would arise during mRNA folding. The resulting strongly folded mRNAs may be more stable, and would tend to accumulate (Supplementary Fig. 9e,f), enabling high protein production rates, and leading to higher protein abundances. In addition, tightly folded mRNA molecules might prompt for slower ribosome progression^10^ (albeit the overall protein production rate is still high, due to the high abundance of the mRNAs). In other words, while ribosomes might be slower on these mRNAs, they contribute to higher levels of protein production, since their stability is higher and they are more abundant.

Slower ribosome progression in turn presumably enables more accurate co-translational protein folding^14,90–92^, thereby leading to stably folded proteins. This is strongly suggested by our data set, since G-/C-ending codons were significantly enriched in folded areas of the proteins (α-helix or β-sheet), while A-/U-ending codons were enriched in unfolded areas (Supplementary Fig. 9g). Thus, the local effects of high G/C-ending codons in some proteins might result in the relative stabilization of these proteins preferentially.

More accurately folded proteins should have smaller solvent-exposed surfaces than unfolded proteins^41^, which would lead to lower rates of chemical damage (*e.g*. oxidation), and thus to longer lifetimes. This reasoning also gives an indication as to why A-/U-ending codons may be preferred in long proteins: to increase the speed of ribosome progression in long transcripts, thereby ensuring their timely translation. An additional possible explanation of some of these effects (at least for mRNAs rich in G- or U-ending codons), could arise from the use of wobble-base pairing during translation. In fact, in some circumstances a tRNA family is being used for both standard (Watson and Crick; G:C) recognition and for wobble (G:U) base-pairing^93,94^. In the case of this non-standard “wobble” base pairing the first nucleotide of the tRNA anticodon (G) can engage with a U in the third position of a codon. Since in metazoans and protozoa this has the effect of reducing protein expression and mRNA stability for the genes that contain high levels of these U-ending codons, this mechanism might in turn promote faster elongation, higher mRNA stability and higher protein levels for mRNAs that are rich of the G-ending codons.

Altogether, these hypothetical scenarios might thus explain how all of the protein homeostasis parameters are interrelated. At the same time, a number of aspects will need further validation. As an example, the helicase activity associated with the ribosome may allow to overcome complex mRNA structures^95,96^, so that the secondary structure of mRNAs may not have as great an influence on the translation efficiency. Another aspect that awaits further clarification is the notion that slow ribosome progression implies better protein folding, since the real situation may be considerably more complex^97,98^.

## Conclusions

Our study contributes to the increasing list of evidence that protein homeostasis parameters are dynamically regulated. We support the idea that codon usage might be used to tune protein turnover, in agreement with scattered evidence that is accumulating in the literature^9,19,90^. At the same time, our work is a step in the direction of shifting the attention on the proteome regulation mechanisms from single cells to entire organisms. This direction will doubtlessly continue to progress, since the expanding palette of “omics” approaches ensures that upcoming studies will benefit from the availability of extended datasets, including protein posttranslational modifications, mRNA modifications and possibly metabolic measurements that will complement the available data.

## Material and Methods

### External datasets

In addition to our mRNA and protein abundance data from mouse^41^, we used several public external datasets for computing the codon correlations to the turnover parameters taken into consideration in this study. These include 15 lifetime data sets, 103 protein abundance data sets, 631 mRNA abundance data sets and 51 data sets of ribosome profiling. The mRNA coding sequences of *E. coli*, *O. Sativa* and *A. thaliana* were obtained respectively from EcoGene 3.0^99^, RAP-DB^100^, and TAIR^101^. For the remaining organisms sequences were obtained from the Ensembl release 84^102^. Protein lengths were acquired from the reviewed protein sequences deposited in Uniprot^103^. Supplementary Dataset 3 summarizes the sources of the datasets that have been included in our analysis.

### Determination of protein lifetimes in the mouse brain *in vivo*

A detailed description of the methods that we have used for determining and validating protein lifetimes *in vivo* is described elsewhere^41^. Briefly, mice have been metabolically pulsed with a Lys_(6)_-SILAC-Mouse labeling diet (^13^C_6_-lysine, Silantes Proteomics, Germany) for different time intervals. Mice were sacrificed, tissues dissected and precise lifetimes were determined through fitting following a thorough evaluation of the pool of available ^13^C_6_-lysines.

### Cell culture and synthetic gene experiments

Syntetic genes were designed *in silico* as detailed in Supplementary Dataset 4 and were ordered at GenScript (USA). Sequences were subcloned in pcDNA3.1(+) plasmids. All final plasmids were confirmed by sequencing. African Green Monkey Fibroblast-like Kidney Cells (COS7) were subcultured according to standard protocols and were incubated at 37 °C in 5% CO_2_. For imaging experiments cells were plated on SensoPlate 96-well glass-bottom plates (Greiner Bio-One) coated with 0.1 mg/ml poly-L-lysine. Cells were transfected in solution with Lipofectamine 2000 (Thermo Fisher Scientific). Before transfection the quantity of DNA was measured in triplicate at the NanoDrop 2000 and confirmed by densitometric analysis on quantitative DNA gel electrophoresis. Using two quantification methods in parallel gave us the same results and allowed us to exclude any possible bias in the measurement of DNA concentration introduced by GC content. As an additional note, since the total length of the plasmid used is >6000 bp, the GC optimization on the final sequence has a very little effect on the total percentage of GC. The overall sequences containing the 0% GC construct have a GC content of ~50%, while the sequence containing the 100% GC constructs have ~54% of GC. For each well of the 96-well plate the same quantity of DNA (330 ± 3 ng) was incubated with 0.66 μl of Lipofectamine. During the imaging experiments, the fluorophore-binding pocket of the protein turnover sensor was blocked overnight with 2 µM SNAP-Cell Block a membrane permeable bromothenylpteridine that binds the SNAP tag^73^, New England Biolabs). Cells were pulsed for 2 h with 1 µM of SNAP-Cell TMR-Star, washed thoroughly and chased in medium containing SNAP-Cell Block, to avoid any possible staining of newly synthetized sensors with the unreacted dyes. Cells were then chased, fixed in 4% buffered PFA, quenched, stained with DAPI, and imaged in PBS at the Cytation 3 cell imaging multi-mode reader (BioTek). In parallel the RNA was extracted from transfected cells using the QIAzol/RNAeasy kit (Qiagen), treated with DNase to avoid any contamination from plasmids, retrotranscribed and analyzed by RT-qPCR using the LightCycle 480 SYBR Green I Master kit (Roche) on a LightCycler 480 system by Roche. To avoid an amplification bias due to the annealing of primers to the optimized sequences, PCR primer pairs were designed for the common 3′-end of the synthetic mRNAs. The following primers (5′ to 3′) were used for amplification: forward, CCCGTTTAAACCCGCTGAT; reverse, ACAGTGGGAGTGGCACCTT.

### Description of the features used for the predictions

For the predictions we used sequence-based features (Supplementary Dataset 1), either alone or in conjunction with Gene Ontology (GO) and protein family information (all features). The rationale for the selection of features was to a large extent driven by postulated theoretical principles and/or observations^55,104^.

We first extracted and compiled relevant information from public databases. In general, the features that we chose can be directly obtained from the gene and protein sequence, or can be calculated from them (*e.g*. secondary structure prediction). In total we selected 710 features (Supplementary Dataset 1). Of these, 11.4% are the percentages of codons or amino acids in a given protein, which could be termed sequence composition features.

We also took into consideration the N-terminal amino acid for each protein^15^, that corresponds to 2.8% of the features. Other potential interesting features that might govern physiological parameters are the protein size, hydrophobicity, and charge, including the isoelectric point, molecular weight, grand average hydropathy (GRAVY), and alipathic index, which make up 1.0% of the total feature set. Since it is probable that the secondary structure of a protein might be important for its biological characteristics, 34.6% of the database consisted of the percentages of codons or amino acids that were predicted to be in β-sheets, α-helixes or coiled-coils. All of these features can be easily calculated from the primary sequence of the proteins (albeit not from the composition alone), and can therefore be termed sequence features.

Gene and protein features such as the percentages of codons and amino acids, N-end amino acids were directly obtained from Universal Protein Resource (UniProt)^103^. Protein features such as the isoelectric point, molecular weight, grand average of hydropathy (GRAVY), and aliphatic indexes were computed with ProtParam^105^ using the perl module (Bio::Tools::Protparam). Secondary structure predictions of proteins were calculated using PSIPRED v3.3^56,106^. The N-terminal amino acid was defined as the amino acid at position 2 of a given protein.

Finally, we added features that describe the localization of a protein or the nature of its domains, resulting in 50.1% non-sequence-derived features from public databases such as GO and PFAM. The biomaRt R package version 2.22.0 was used to extract GO, protein, and protein family information. In more detail, we extracted GO information for the categories ‘biological process’, ‘molecular function’, and ‘cellular component’ from the GO database^107^. Since GO contains a large number of terms we limited GO-derived features to the ones that can be found in at least in 10 proteins. Protein family information was extracted from PFAM^108^. It is important to note that we excluded certain features in some predictions, since they were directly related or equal to the response variable. As an example, we have excluded the ‘protein length’ as a feature when predicting protein length.

### Modeling and predictions

Our prime interest was to predict protein characteristics (*e.g*. protein half-life) with as high accuracy as possible, using a set of features (predictors). The importance of the predictors from the models can provide biological insights on how (strongly) they influence biological characteristics. Based on this information we can then modify genes or proteins for a defined biological outcome (*e.g*. half-life). The first step was to generate a high-quality database of predictors and response variables of interest, as described in the previous section for the response variables. Further crucial steps in model building were data pre-processing, model selection, model optimization, and quality control. The remaining paragraphs of this section will give a general description of these steps.

#### Pre-processing

All predictors were centered and scaled to avoid predictor selection bias. In addition, models were built with and without filtering for co-linear predictors, predictors that are linear combinations of each other, or close to zero-variance predictors to avoid model optimization problems. In general we did not see performance or feature selection differences for models built with filtered or non-filtered data, and we therefore do not report the filtered model performances in this manuscript, beside what shown in Supplementary Fig. 2.

#### Model selection

The next crucial step is the model selection, which defines the machine-learning algorithm best suited to the task. The first choice is easy: do we choose a regression or classification algorithm? Or, in other words, is the response variable continuous or categorical? In our case, all responses are real (${\mathbb{R}}$) (*e.g*. protein turnover) or natural (${\mathbb{N}}$) numbers (*e.g*. mRNA expression), which are best represented by a regression model.

The second consideration is if we choose a linear regression model or a non-linear regression model, or, in other words, whether our predictors are linearly or non-linearly related to the response variable. For sequence data, each predictor was fit to the response variable using univariate first and fifth degree polynomial regression and the RMSE was measured. For most response variable several predictor combinations polynomials of degree 1 (linear models) fit the data best or almost as good as fifth-order polynomial functions, pointing towards linear relationships of the predictors to the response variables. Therefore, we chose to use two strictly linear regression approaches, elastic-net^109^ (glmnet) and FoBa^53^. Although it seemed that the individual response variables have a linear relationship to the respective response variables, it was not clear if combinations of predictors might not have a non-linear relationship that explains the response variable better. In order to capture linear and potential non-linear relationships we also used the random forests (RF) approach^54^.

Moreover, our data contained in general many predictors (p_min_ = 71, p_max_ = 710) as compared to the observations (n_min_ = 1325, n_max_ = 4560), which made it easy to overfit the model (performance overestimation), and made the model hard to interpret (model complexity). In addition, we expected that only few predictors might carry valuable information in predicting the response variable, a condition referred to as ‘sparsity in representation’ in the literature. Several algorithms have been developed to efficiently learn a sparse target function, among them being popular tools such as lasso^110^, FoBa^53^, and elastic-net^109^. All of these algorithms have in common that they simultaneously solve for the two main interests: predictor selection (selecting the basis functions with non-zero coefficients) and estimation accuracy (reconstruction of the target function from noisy data). In other words, the algorithms optimize for a simple model that is easy to interpret biologically while being very accurate.

#### Model optimization

Given the relatively large number of observations (at least for biological and medical machine learning) and the algorithm’s inherent feature selection during cross-validation, we decided to optimize models using a classical training – cross-validation – testing approach. In detail, data was split into a test set (20% of the total dataset), which was only used for the performance evaluation of the final model as reported in the main text, and a training – cross-validation set that encompassed the remaining 80% of the dataset. Model hyper-parameters and model sparsity (predictor selection) were optimized by minimizing the root-mean-square error (RMSE) during the 10-fold cross-validation with 10 different initializations (repetitions), splitting the training – cross-validation set into balanced 90% training and 10% cross-validation sets. Subsequently, models were optimized by minimizing the RMSE on the full training – cross-validation set and final model performance was assessed on the test set.

#### Quality control

Proper model selection and optimization was assessed with a multitude of metrics and plots. We used model selection plots to assess the training and cross-validation error against the degree of model complexity, a strictly convex process for our learning algorithms. Whereas high training and cross-validation errors would signify high bias (under-fitting), low training and high cross-validation errors would indicate high variance (over-fitting). In general, we did not observe over-fitting, which is due to our careful model optimization, but cannot exclude that some models, especially for mRNA abundance, are under-fitted due to missing predictors such as promoter and enhancer region information. In addition, the prediction of protein length is non-linearly dependent from the predictors, and the strictly linear elastic-net and FoBa algorithms are in consequence biased (systematic error due to wrong model selection).

An additional diagnostic we used are learning curves, plotting increasing sets of observations against training and cross-validation errors. Low training error and high cross-validation error signify high variance and could most probably be optimized by increasing the amount of observations. High training and cross-validation errors indicate high bias and an increase in the number of observations would most probably not enhance model performance, whereas usage of a different model or addition of predictors could help. Overall, we did observe convergence of training and cross-validation errors with increasing observations for our models, indicating that models are neither affected by high variance nor by bias, and that the addition of further observations might not result in dramatic increases of model performance.

To assess the stability of the models we plotted the RMSE and *r*^2^ of all cross-validation folds. While high variability would indicate model instability, which could be due to unbalanced data partitions or to highly variable data, we observed mostly low variance for cross-validation folds. Furthermore, cross-validation and test RMSE and *r*^2^ values were in almost all instances comparable (emanate from same distribution), indicating model stability and generalizability. Model stability is also reflected in the near equal performance of the elastic-net, FoBa, and RF algorithms in most cases. To assess the importance of specific predictors we calculated feature importances for each model and scaled the resultant values between 0 and 100, to make them comparable between the different algorithms. Unscaled feature importances for FoBa and elastic-net models represent the coefficient estimates divided by the standard deviation of the coefficients (t-statistic), as estimated from a 30-fold cross-validation on the training – cross-validation set. Unscaled RF feature importances represent the mean decrease in node impurity. For the actual computations we used the R caret package^111^ with the packages glmnet (elastic-net), FoBa, and RF, complemented by in house scripts.

### Code Availability

The code used in this work is available from the corresponding authors upon reasonable request.

### General data analysis and statistics

Protein information was retrieved from The Universal Protein Resource (UniProt Consortium^112^). All analyses were performed with the help of Matlab (The Mathworks Inc., Natick, MA, USA) and SigmaPlot software (Systat Software), using self-written routines.

The codon usage (the frequency with which a particular codon appears for each 1000 codons in the mouse mRNA-ome) was taken from the publicly available Kazusa database (http://www.kazusa.or.jp/e/index.html). Image analysis (Fig. 4) was performed using self-written routines in Matlab. Briefly, the cell nuclei were selected in the DAPI channel by applying an empirically derived threshold, which was identical for all of the images. The threshold procedure generated region-of-interest masks for all of the nuclei, which following segmentation were expanded to include the extranuclear region of the cells. The fluorescence intensity in the channel corresponding to the dye used to label the protein turnover sensors (rhodamine channel) was then determined in the masks (typically 3000–4000 masks per time point and experiment, for a total of >10,000 cells analyzed for each experiment). These values were then averaged for each of the experiments. To make sure that in our imaging experiments were carried out in the range of accurate detection we performed a calibration experiment where we measured serial dilutions of the SNAP-Cell TMR-Star that was used in our SNAP-tag labeling. Briefly, the TMR-Star was diluted in 25 µl of PBS and imaged on the Cytation 3 high content microscope on 96-well plates with exactly the same settings that were used for imaging the cells in our turnover experiments (see also Supplementary Fig. 7).

## Electronic supplementary material

Supplementary file

Dataset 1

Dataset 2

Dataset 3

Dataset 4

## Data Availability

The authors declare that the data supporting the findings of this study are available or, as stated elsewhere, will be given upon reasonable request.
