# Supplementary material for: The codon sequences predict protein lifetimes and other parameters of the protein life cycle in the mouse brain
Source: Sci Rep. 2018 Nov 15;8:16913. doi: 10.1038/s41598-018-35277-8 (PMC6237891; doi:10.1038/s41598-018-35277-8)
Supplement: Supplementary file 1 — Supplementary file [file 41598_2018_35277_MOESM1_ESM.pdf]

Supplementary Information

of the manuscript:

The codon sequences predict protein lifetimes and other parameters of the protein life cycle in the mouse brain

Author list:

Sunit Mandad[1,2], Raza-Ur Rahman[3,11], Tonatiuh Pena Centeno[3], Ramon O. Vidal[3], Hanna Wildhagen[1], Burkhard Rammner[1], Sarva Keihani[1], Felipe Opazo[1,4], Inga Urban[5], Till Ischebeck[6], Koray Kirli[7], Eva Benito[8], André Fischer[8,9], Roya Y. Yousefi[10], Sven Dennerlein[10], Peter Rehling[10], Ivo Feussner[6], Henning Urlaub[2], Stefan Bonn[3,11,12]*, Silvio O. Rizzoli[1,4]*, Eugenio F. Fornasiero[1]*

Author information:
[1] Department of Neuro- and Sensory Physiology, University Medical Center Göttingen, Cluster of Excellence Nanoscale Microscopy and Molecular Physiology of the Brain, Göttingen, Germany.
[2] Department of Clinical Chemistry, University Medical Center Göttingen, Göttingen, Germany, and Bioanalytical Mass Spectrometry Group, Max Planck Institute of Biophysical Chemistry.
[3] Laboratory of Computational Systems Biology, German Center for Neurodegenerative Diseases (DZNE) Göttingen, Germany.
[4] Center for Biostructural Imaging of Neurodegeneration (BIN), Göttingen, Germany.
[5] Genes and Behavior Department, Max Planck Institute of Biophysical Chemistry, Göttingen, Germany.
[6] Department of Plant Biochemistry, Albrecht-von-Haller-Institute, Georg-August-University, Göttingen, Germany.
[7] Department of Cellular Logistics, Max Planck Institute for Biophysical Chemistry, Göttingen, Germany.
[8] Laboratory of Epigenetics in Neurodegenerative Diseases, German Center for Neurodegenerative Diseases (DZNE) Göttingen, Germany.
[9] Department of Psychiatry and Psychotherapy, University Medical Center Göttingen, Göttingen, Germany.
[10] Department of Cellular Biochemistry, University Medical Center Göttingen, Göttingen, Germany and Max Planck Institute for Biophysical Chemistry.
[11] Institute of Medical Systems Biology, Center for Molecular Neurobiology (ZMNH), University Medical Center Hamburg-Eppendorf (UKE).
[12] German Center for Neurodegenerative Diseases (DZNE) Tübingen, Germany.

*Correspondence to: Stefan Bonn (sbonn@uke.de), Silvio O. Rizzoli (srizzol@gwdg.de) and Eugenio F. Fornasiero (efornas@gwdg.de).

SUPPLEMENTAL INFORMATION

Supplementary Datasets (available as excel files)**:**
Supplementary Dataset 1: List of features and relative importances of the RF predictions
Supplementary Dataset 2: Comparison of r and r^2 values for all RF predictions
Supplementary Dataset 3: References for the external databases
Supplementary Dataset 4: Synthetic genes

SUPPLEMENTARY FIGURES:
Supplementary Figure 1: Protein homeostasis parameters are conserved
Supplementary Figure 2: Models and predictions
Supplementary Figure 3: N-terminal degrons
Supplementary Figure 4: Correlations between different protein homeostasis parameters
Supplementary Figure 5: Sequence correlations
Supplementary Figure 6: The codon behavior in relation to turnover parameters is conserved
Supplementary Figure 7: Evaluation of signal linearity in the imaging setup used
Supplementary Figure 8: Calmodulin has a half-life of ~6h as measured with analogous approaches
Supplementary Figure 9: Additional data supporting the hypothetical scenario introduced in Fig. 6

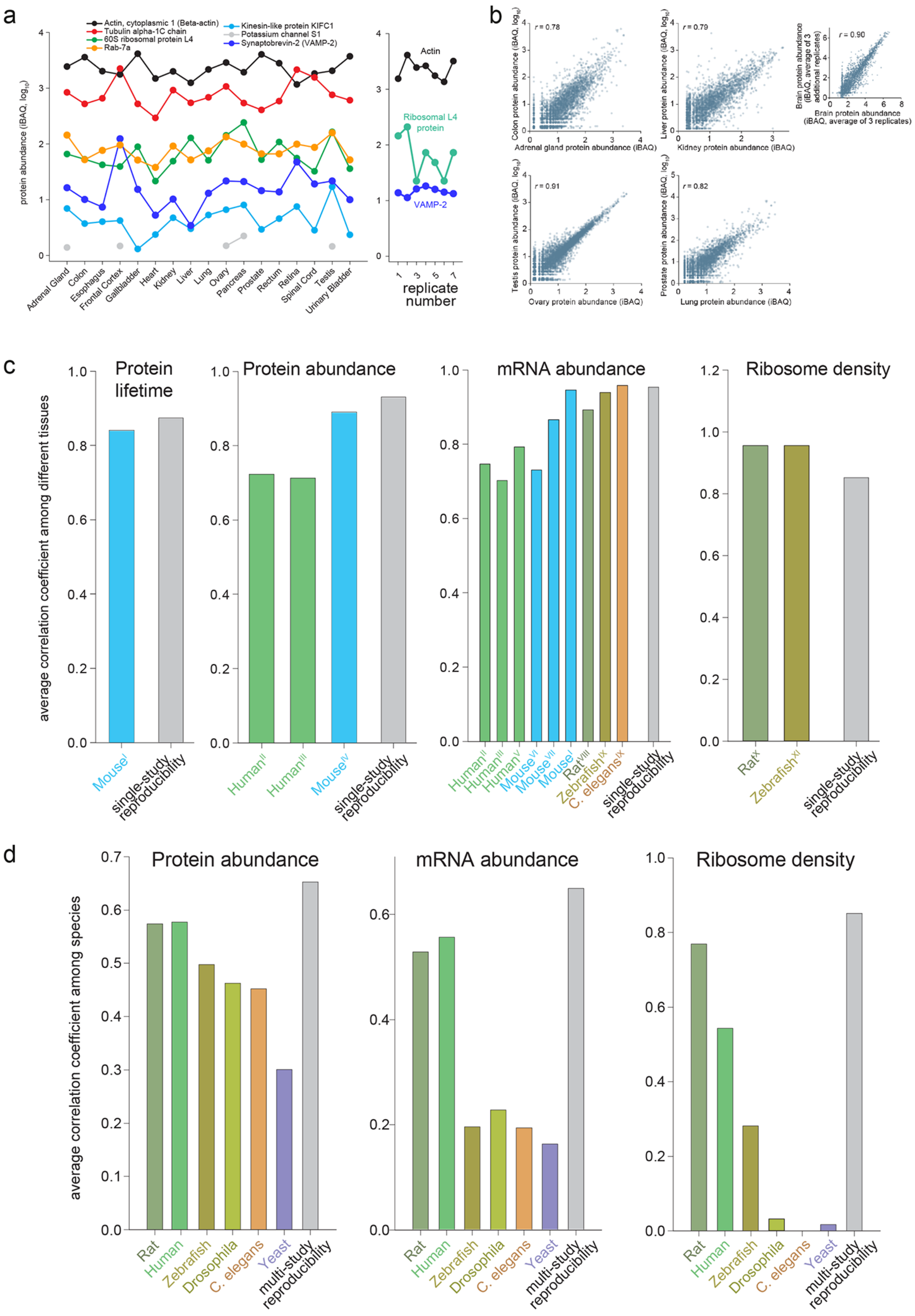

Legend in the following page

Supplementary Figure 1 Protein homeostasis parameters are conserved among tissues and conditions (**a**) Examples of protein amounts compared between different human organs and from different technical replicates (inset on the right). The levels of the proteins do vary among tissues, albeit not enormously: for example, abundant proteins are relatively abundant in all tissues. (**b**) Protein amounts compared between different human organs, or among technical replicates (inset on the right). Part of the variation is due to technical replicates. (**c**) Correlation coefficient among different tissues. The data used for the analysis derives from different studies. In detail: I[1]; II[2]; III[3]; IV[4]; V[5] ; VI[6]; VII[7]; VIII[8]; IX[9]; X[10] and XI[11]. (**d**) Correlation coefficient among different species. The data used was obtained from several different studies and it is summarized in Supplementary Dataset 3.

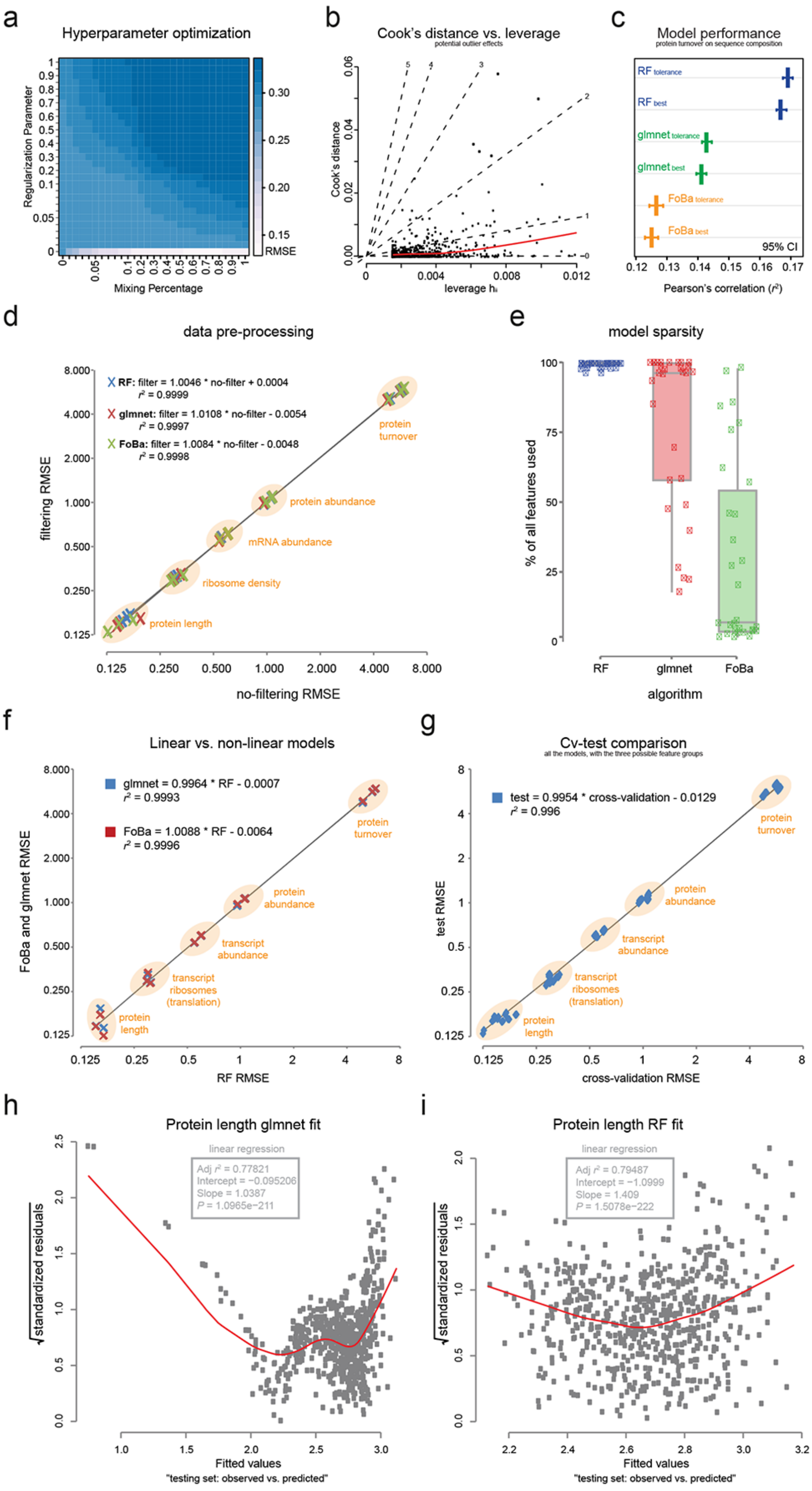

Legend in the following page

Supplementary Figure 2 Models and predictions. (**a**) Hyper-parameter optimization. For each model hyper-parameters such as the mixing percentage and regularization (glmnet) were optimized for the lowest RMSE (white regions) during cross-validation. Parameters were then estimated based on the whole cross-validation set and final model performance was assessed on the test set. (**b**) Outlier detection. Data that contains outliers, in other words observations that are extreme and potentially biased, can distort the accuracy of a regression analysis. To estimate the impact of potential outliers the leverage and Cook's distance were plotted for each fit, and observations with high leverage and Cook's distance were inspected more closely. Altogether, we did not observe outliers that would warrant removal, further consolidating the robustness of the models and predictions. (**c**) Cross-validation performance of RF, glmnet, and FoBa models predicting protein turnover based on sequence composition features. Shown are the mean Pearson's correlation and 95% confidence intervals (CI) for the best model and the tolerance model (within 2% of best model RMSE). (**d**) Filtering for linearly dependent or near zero variance features does not affect model performance. Shown are the RMSEs of all models (RF in blue, elastic-net in red, FoBa in green) built with (filtering RMSE) or without (no-filtering RMSE) filtering for co-linear dependencies or near zero variance predictors (features). (**e**) Model sparsity overview. Box-and-whisker representation of the percentage of features used for all response – feature-set comparisons. (**f**) Comparison of linear and non-linear models. (**g**) Cross-validation and test RMSEs for all models and response – feature-set comparisons are highly similar, providing evidence for good model generalization. (**h, i**), Protein length does not show a linear correlation with the feature-sets used. (**h**) A strictly linear glmnet prediction of the logarithm of the protein length shows a strong bias for short and long proteins whereas (**i**) RF predictions of the logarithm of the protein length shows a good fit.

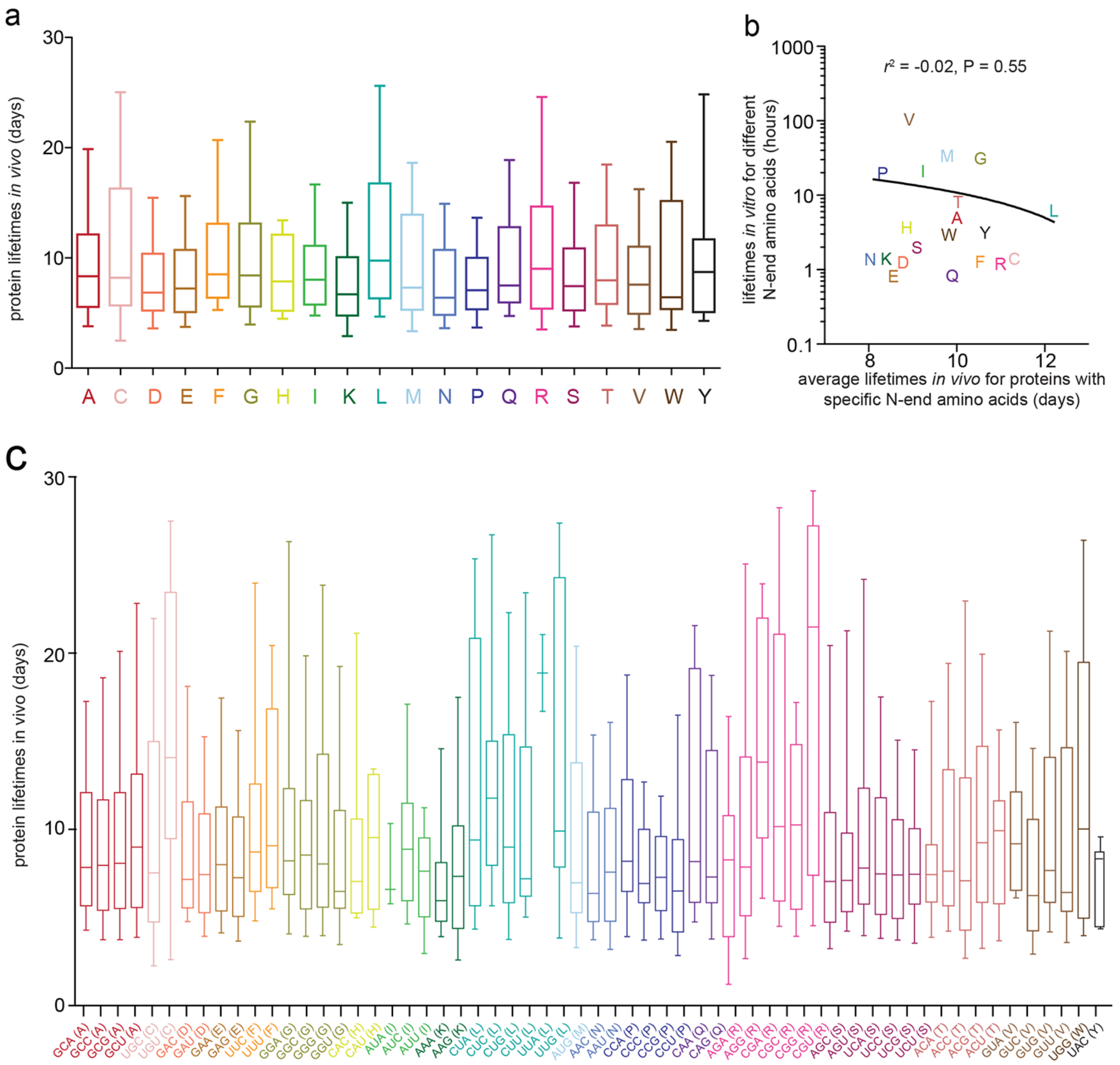

Supplementary Figure 3 N-terminal degrons and N-terminal codon usage have a limited influence on protein lifetimes. (**a**) Box-and-whisker representation of the distribution of protein lifetimes (from[1] with respect to the first amino acid that follows the N-terminal methionine. An ANOVA test revealed only a handful of significant differences, with proteins containing N-terminal Leucines being significantly more stable than those containing D, E, N, K, S, V and P (p<0.001). The relevance of this observation is unclear. The fact that the predictive models have not selected this feature suggests that this is probably redundant with respect to other features. (**b**) Scatter plot of protein lifetimes from the mouse brain *in vivo* against the lifetimes determined in mammalian reticulocytes for N-terminal degrons *in vitro*, also known as the N-end rule (respectively from[1] and[12]). There is no correlation between the *in vivo* measurements and the lifetime observed *in vitro* in reticulocyte experiments, suggesting that *in vivo* the effects of the N-end rule are probably overruled by other factors, an therefore the N-end rule has virtually no correlation with the protein lifetimes measured *in vivo* (**c**) Same as panel a but plotting single codons separately. The ANOVA analysis indicates that the UUG codons for Leucine follow the same trend observed with the amino acids (p<0.01 against GAG (E), CCC (P), CCG (P), UCG (S) and UCU (S)).

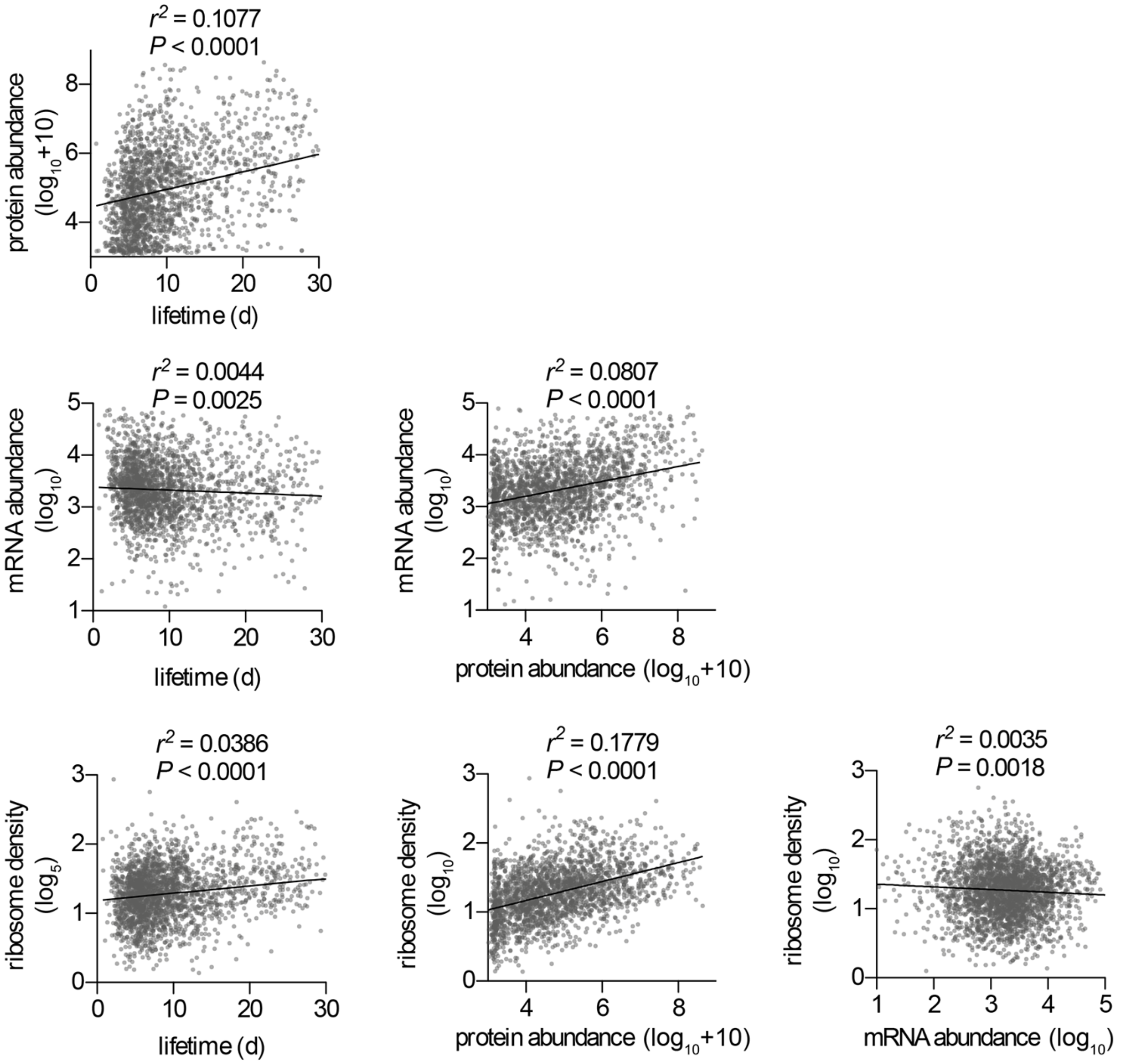

Supplementary Figure 4 Limited correlations can be found between different protein homeostasis parameters in the mouse brain. We measured protein abundances in the mouse brain using iBAQ[13] and mRNA abundances by whole transcriptome shotgun sequencing[14]. The ribosome density values were obtained from a published study[15].

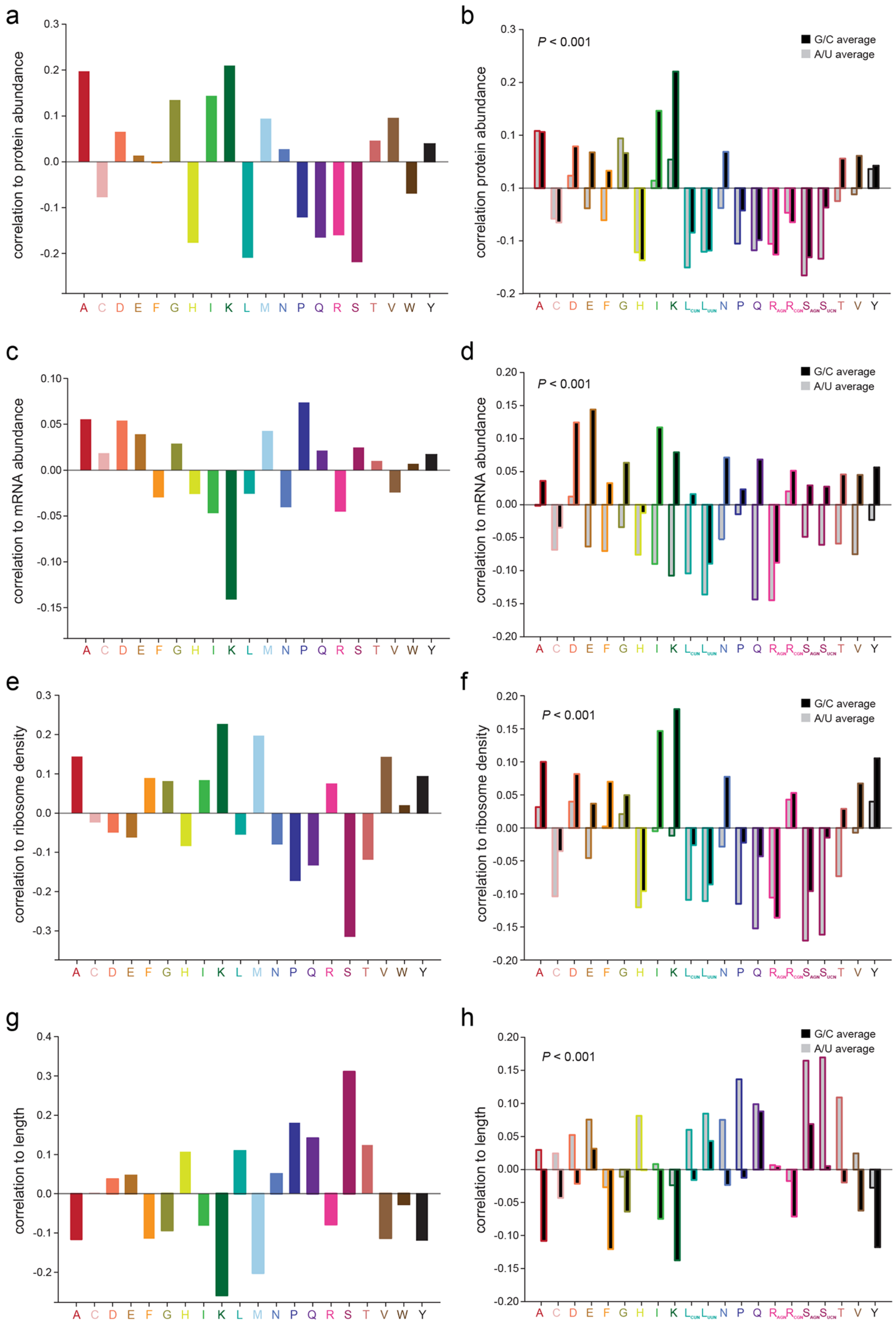

Legend in the following page

Supplementary Figure 5 Sequence correlations to protein and mRNA abundances, to the ribosome density, and to the protein length. We calculated the amino acid and codon correlation coefficients to these parameters, in the same fashion we have done in Fig. 1b and 1e for the protein lifetime. The plots show the coefficients of the amino acids (left panels), and the coefficients of the codons, averaged for the G-/C- or A-/U-ending codons of each amino acid (right panels). (**a,b**) Protein abundance. This parameter was determined in our brain cortex samples by iBAQ (see Methods for details). (**c,d**) mRNA abundances, as determined in brain cortex samples[1] (**e,f**) The ribosome density on mRNA molecules, as measured by Gonzales and collaborators in the mouse brain[15] (**g,h**) The protein length, as annotated on the mouse UniProt database. For proteins with multiple splicing isoforms, the most abundant isoform was taken into consideration. The results were then averaged, and the coefficients to these averages are plotted. For the codon analysis, we tested statistically whether the coefficients of the G-/C- or A-/U-ending codons were different, using t-tests (n = 21 sets of codons). Indeed, the coefficients of the G-/C-ending codons were significantly larger (more positive) than those of the A-/U-ending codons for the protein and mRNA abundances and for the ribosome density. The opposite was observed for the protein length. The *P* values are indicated on the graphs.

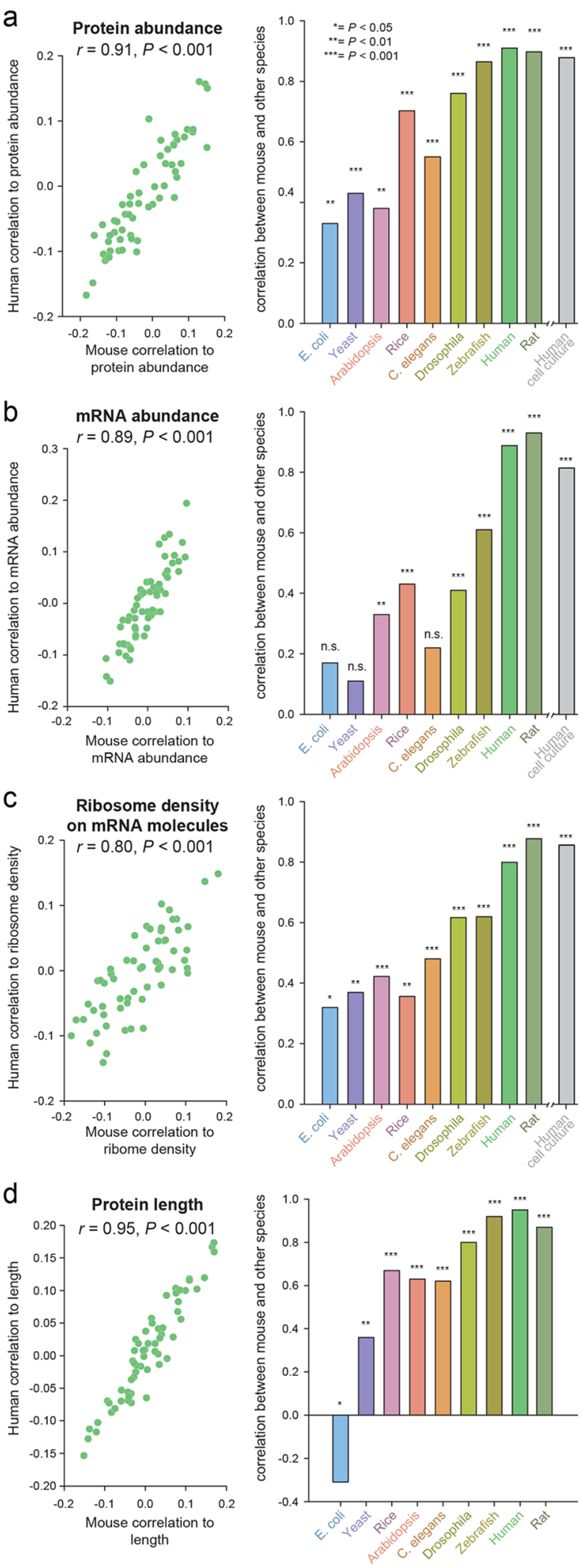

Legend in the following page

Supplementary Figure 6 The codon behavior in relation to turnover parameters is conserved through evolution. (**a-d**) We used a total of 800 data sets from the literature to investigate the correlation between the mouse codon coefficients and the coefficients from different organisms (refer to Supplementary Dataset 3 for details concerning the databases taken into consideration). In detail we evaluated the protein abundance (**a**), the mRNA abundance (**b**), the ribosome density on mRNA transcripts (**c**) and the protein length (**d**) for the following organisms: bacteria (*Escherichia coli*), yeast (*Saccharomyces cerevisiae*), rice (*Oryx sativa*), mouse-ear cress (*Arabidopsis thaliana*), nematode (*Caenorhabditis elegans*), fruit fly (*Drosophila melanogaster*), zebrafish (*Danio rerio*), human (*Homo sapiens*) and rat (*Rattus norvegicus*). The panels on the left are scatter plots of the mouse codon coefficients against the coefficients arising from human tissues. The scatter plots serve as examples for this analysis, and each dot represents a codon. We calculated the Pearson correlation between the human and mouse codon coefficients (*r*, shown on the plots, along with the *P* value). The *r* serves as an indication for how similar the codon behavior from the mouse is to that from the human. The Pearson correlation *r* values were also obtained between the mouse codon coefficients and those from all other species, and were plotted in the bar graphs. The high *r* values between the results obtained in mouse and in other vertebrates (human, rat, zebrafish) show that codons relate in the same fashion to the different turnover parameters in all of these organisms (*e.g.*, the same codons are linked to high protein abundance in mouse, human, rat and zebrafish). The similarity is lower for other organisms, but most correlations are nevertheless significant (the *P* values of the Pearson's correlation coefficients are shown above the bars; *n* = 61 codons for all bars). The values calculated for human cell lines are also represented, and indicate that these correlations are maintained in immortalized cells (gray bars; no gray bar is shown for the protein length, since the protein lengths in human tissues and in human cell cultures are identical – only one bar, for the human tissues, is shown).

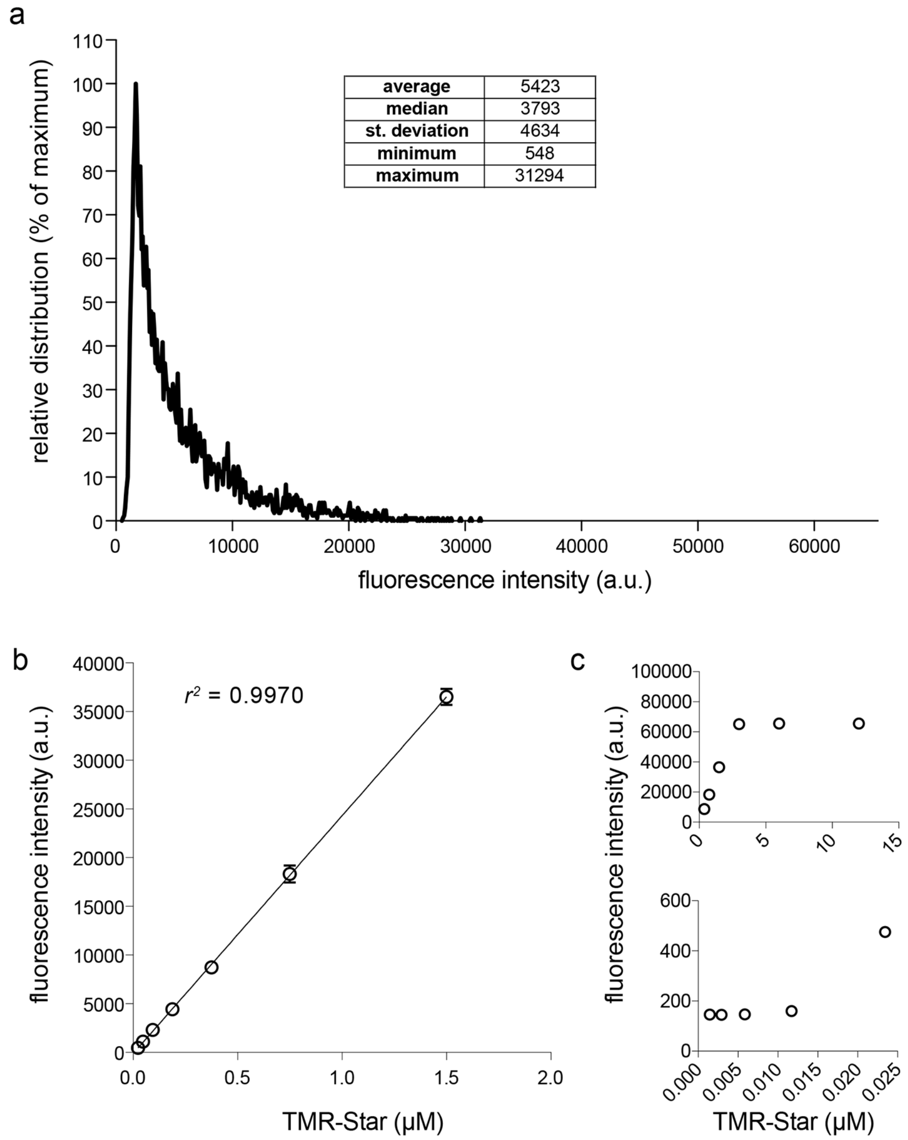

Supplementary Figure 7 Evaluation of signal linearity in the imaging setup used for the determination of protein lifetimes. (**a**) Distribution of fluorescence intensities in the imaging experiments used to evaluate protein turnover (Fig. 4b and Fig. 5a-d). All detected cells have a signal intensity ranging from 548 to 31294 arbitrary units. (**b**) Detection of serially-diluted the SNAP-Cell TMR-Star dye with the same settings used in the imaging experiments summarized in panel **a**. The signal shows a virtually perfect linearity in the range where all detected cells are distributed. (**c**) The signal is saturated for values higher than 2^{16} a.u. (65536) and loses its linearity for values under ~200 a.u., where the fluorescence of the detected dye has probably the same magnitude of the auto-fluorescence detected by the setup (background).

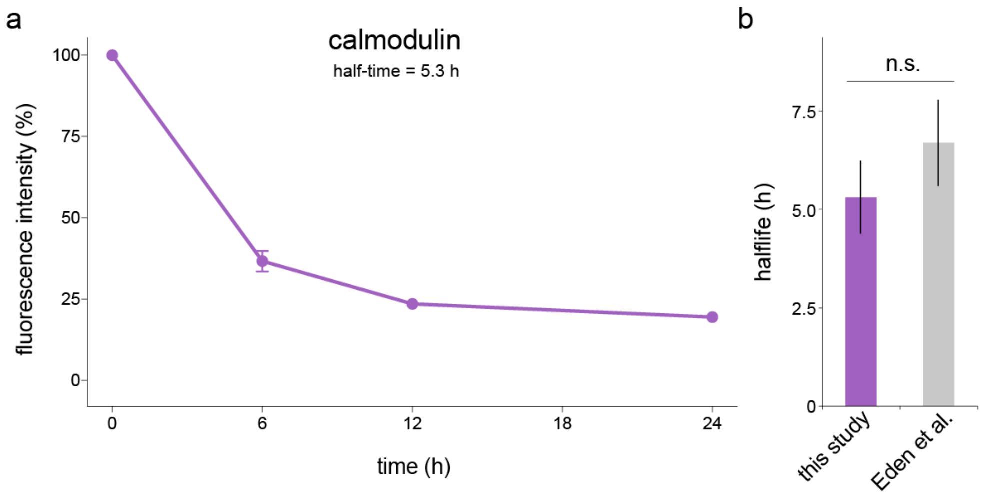

Supplementary Figure 8 Calmodulin has a half-life of ~6h as measured with an analogous imaging approach[16]**.** (**a**) A calmodulin-SNAP turnover sensor was created and transfected in COS7 cells. Experiments were carried out as in **Fig. 4** and **Fig. 5**. Briefly, the graph represents the chase following a 2h pulse of the new protein (synthesized just during the pulse). For every chase time, cells were fixed, imaged with a high content microscope and analyzed in an automated fashion. Each dot in the graph represents the average of three separate experiments with SEM (n = 3) and shows the results from >2’000 cells analyzed per condition. (**b**) Lifetime (expressed as $t_{1/2}$) calculated from the values from panel **a** compared with the lifetime reported in Supplementary Table 4 from Eden *et al*[16]. The two calculated lifetimes are not significantly different. The fact that the two lifetimes are not exactly the same might be due to the differences in cell types and/or in the fusion tag used (respectively SNAP-tag for our experiments and GFP for the bleach-chase approach used by Eden and collaborators).

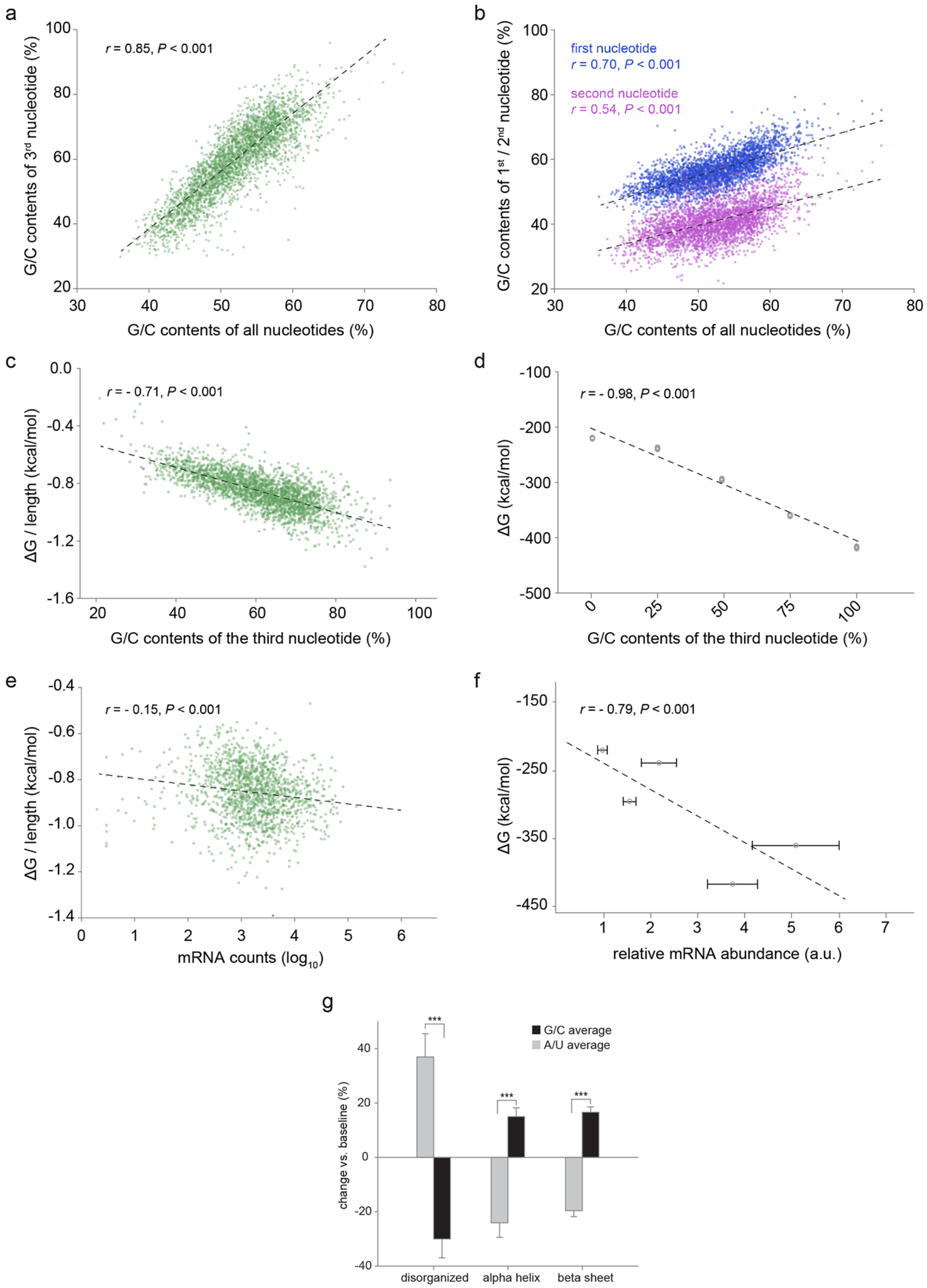

Legend in the following page

Supplementary Figure 9 Additional data supporting the hypothetical scenario introduced in Figure 6**.** (**a**) The overall G/C contents of the mouse brain mRNAs correlate well to the G/C contents at the third position of their codons. (**b**) Interestingly, the G/C contents at the first and at the second position of codons are correlated to the overall G/C contents of mRNAs, but to a lesser extent than for the third nucleotide, implying that the latter is the most important nucleotide in determining the overall G/C contents. (**c**) The G/C contents at the third nucleotide is negatively correlated to the estimated free energy (ΔG) of mRNAs (normalized for mRNA length). Since lower free energies correspond to more stably folded structures, higher G/C contents at the third nucleotide implies more stably folded mRNAs. (**d**) The same was observed in the case of our synthetic genes (from Figure 6). (**e** and **f**) Lower free energies (more stably folded mRNA structures) correlate with more abundant mRNAs both in our mouse brain dataset (**e,** determined by next generation sequencing) and in our synthetic gene subset (**f,** determined by RT-qPCR). (**g**) G-/C-ending codons are more frequently used to code for structured protein domains**.** In detail, the analysis of the percentage of G-/C- or A-/U-ending codons in the vicinity of regions with defined protein structures revealed that disorganized protein regions have a prominent increase of A-/U-ending codons, while more structured regions, containing alpha helices and beta sheets, are characterized by G-/C-ending codons. The data were obtained from the secondary structure calculations used in Figure 3, which provide the probable secondary structure at each amino acid. Only regions in which the particular structure (disorganized, alpha helix or beta sheet) was found with high probability over several adjacent amino acids were analyzed, to avoid the analysis of uncertain structures.

Supplementary References

1. Fornasiero, E. *et al.* Precisely measured protein lifetimes in the mouse brain reveal differences across tissues and subcellular fractions. *Nat. Commun.* (2018). doi:10.1038/s41467-018-06519-0
2. Kim, M.-S. *et al.* A draft map of the human proteome. *Nature* **509,** 575–581 (2014).
3. Wilhelm, M. *et al.* Mass-spectrometry-based draft of the human proteome. *Nature* **509,** 582–7 (2014).
4. Sharma, K. *et al.* Cell type- and brain region-resolved mouse brain proteome. *Nat. Neurosci.* **18,** 1819–31 (2015).
5. Lu, T. *et al.* REST and stress resistance in ageing and Alzheimer's disease. *Nature* **507,** 448–54 (2014).
6. Zapala, M. a *et al.* Adult mouse brain gene expression patterns bear an embryologic imprint. *Proc. Natl. Acad. Sci. U. S. A.* **102,** 10357–10362 (2005).
7. Kadakkuzha, B. M. *et al.* Transcriptome analyses of adult mouse brain reveal enrichment of lncRNAs in specific brain regions and neuronal populations. *Front. Cell. Neurosci.* **9,** 63 (2015).
8. Yu, Y. *et al.* A rat RNA-Seq transcriptomic BodyMap across 11 organs and 4 developmental stages. *Nat. Commun.* **5,** 3230 (2014).
9. Levin, M. *et al.* The mid-developmental transition and the evolution of animal body plans. *Nature* **531,** 637–641 (2016).
10. Ori, A. *et al.* Integrated Transcriptome and Proteome Analyses Reveal Organ-Specific Proteome Deterioration in Old Rats. *Cell Syst.* **1,** 224–237 (2015).
11. Housley, M. P. *et al.* Translational profiling through biotinylation of tagged ribosomes in zebrafish. *Development* **141,** 3988–93 (2014).
12. Gonda, D. K. *et al.* Universality and structure of the N-end rule. *J. Biol. Chem.* **264,** 16700–16712 (1989).
13. Schwanhäusser, B. *et al.* Global quantification of mammalian gene expression control. *Nature* **473,** 337–42 (2011).
14. Holt, R. A. & Jones, S. J. M. The new paradigm of flow cell sequencing. 839–846 (2008). doi:10.1101/gr.073262.107.cell
15. Gonzalez, C. *et al.* Ribosome Profiling Reveals a Cell-Type-Specific Translational Landscape in Brain Tumors. *J. Neurosci.* **34,** 10924–10936 (2014).
16. Eden, E. *et al.* Proteome half-life dynamics in living human cells. *Science (80-.).* **331,** 764–768 (2011).
